# Identification and Characterization of Antimicrobial Peptides From Butterflies: An Integrated Bioinformatics and Experimental Study

**DOI:** 10.3389/fmicb.2021.720381

**Published:** 2021-08-26

**Authors:** Min Wang, Ziyue Zhou, Simin Li, Wei Zhu, Xianda Hu

**Affiliations:** ^1^Beijing Hospital, National Center of Gerontology, Beijing, China; ^2^Institute of Geriatric Medicine, Chinese Academy of Medical Sciences, Beijing, China; ^3^Peking Union Medical College Hospital, Chinese Academy of Medical Sciences and Peking Union Medical College, Beijing, China; ^4^Stomatological Hospital, Southern Medical University, Guangzhou, China; ^5^Institute of Stem Cell and Regenerative Biology, College of Animal Science and Veterinary Medicine, Huazhong Agricultural University, Wuhan, China; ^6^Key Laboratory of Agricultural Animal Genetics, Breeding, and Reproduction of Ministry of Education, Huazhong Agricultural University, Wuhan, China; ^7^Beijing Tibetan Hospital, China Tibetology Research Center, Beijing, China

**Keywords:** antimicrobial peptide, butterfly, bioinformatics, identification, characterization

## Abstract

Butterflies represent one of the largest animal groups on Earth, yet antimicrobial peptides (AMPs) of this group are less studied in comparison with their moth counterparts. This study employed an integrated bioinformatics approach to survey natural AMPs from publicly available genomic datasets. Numerous AMPs, including cecropins, defensins, and moricins, were identified and subsequently used as templates for the design of a series of synthetic AMPs that mimicked the naturally occurring sequences. Despite differing biological effects among the various sequences, the synthetic AMPs exhibited potent antibacterial and antifungal activities *in vitro* and *in vivo*, without inducing hemolysis, which implied their therapeutic potential in infectious diseases. Electron and confocal fluorescence microscopies revealed that the AMPs induced distinct morphological and biophysical changes on microbial cell membranes and nuclei, suggesting that the antimicrobial effects were related to a mechanism of membrane penetration and nucleic acid binding by the peptides. In conclusion, this study not only offers insights into butterfly AMPs but also provides a practical strategy for high-throughput natural AMP discoveries that will have implications for future research in this area.

## Introduction

Infectious diseases were a major problem in human history until the development of various efficacious antibiotics that target the causative microorganisms. However, to adapt an aphorism of Friedrich Nietzsche, that which does not kill them makes them stronger, consequently, antimicrobial resistance emerged thereafter and has become a significant threat to public health worldwide. Declining numbers of new antibiotic candidates has further worsened concern about the shortage of antibiotics in the near future, and development of new classes of antimicrobial agents is therefore urgently required (Roope et al., 2019).

Antimicrobial peptides (AMPs) are a heterogeneous group of small amino acid molecules that are evolutionarily conserved in the genome and ubiquitously produced throughout the kingdoms of life to combat microbial infections (Unckless and Lazzaro, 2016). As effector molecules of the innate host defense, AMPs are vital to all living organisms, especially simpler organisms that only depend on innate and humoral immunity to prevent the onset of infection (Jasper and Bohmann, 2002). In recent decades, AMPs have received considerable attention as a promising group of molecules due to their unique mechanism of rapid physical disruption of microbial membranes, although this dogma is now being challenged, as alternative targets have also been reported (Manniello et al., 2021). This mechanism is preferable for suppression of the deteriorating resistance problem because microbes are eliminated regardless of antibiotic sensitivity or resistance, and also AMPs are not prone to inducing resistant mutants (Zharkova et al., 2019). Furthermore, other desirable properties such as broad-spectrum activity and low host toxicity make AMPs appropriate alternatives to antibiotics (Hazam et al., 2019).

To date, numerous AMPs have been researched as drug candidates, and many are currently in clinical trials, the majority of which are naturally derived (Greber and Dawgul, 2017). As one of the most diverse and abundant orders of animals on Earth, Lepidoptera, which comprises butterflies and moths, is a likely major source of natural AMPs (Badapanda and Chikara, 2016). Among the order Lepidoptera, Papilionoidea (butterflies), which comprises typical butterflies (Lycaenidae, Nymphalidae, Papilionidae, Pieridae, and Riodinidae), skippers (Hesperiidae), and moth-butterflies (Hedylidae) (Kawahara and Breinholt, 2014), is one of the most speciose superfamilies, but there are limited studies on AMPs of this superfamily. However, most available genomic datasets of lepidopteran species belong to the butterflies, which are characterized by their relatively small genome size and relatively low repeat content when compared with other eukaryote genomes (Triant et al., 2018). Therefore, identification of the transcripts and gene-encoded peptidyl sequences from butterfly genomes is a feasible approach to investigate whether high-throughput sequencing data are a potential repository for natural butterfly AMP sequences.

In this study, an integrative bioinformatics analysis was conducted to discover naturally occurring AMPs from butterfly genomes. Based on the sequence and structural characteristics of these butterfly AMPs, a series of synthetic analogs were designed and synthesized. The antimicrobial activities and mechanisms of these synthetic molecules were studied and subsequently discussed (Supplementary Figure 1).

## Materials and Methods

### *In silico* Screening of Butterfly Antimicrobial Peptides

Complete genome sequences of Papilionoidea species available in the Gene Expression Omnibus (GEO) database were downloaded (Supplementary Table 1). *Ab initio* gene predictions were performed using Augustus 3.3.1^1^ (Stanke et al., 2008) trained on the *Heliconius melpomene* genome model (parameters: augustus --softmasking = true --species = heliconius_melpomene1 genome.fasta), and complete predicted open reading frames (ORFs) of up to 400 amino acids were retained. The predicted encoded amino acid sequences were aligned against AMP sequences obtained from the RefSeq protein database^2^ using BLASTp (O’Leary et al., 2016), with the highest scoring homologies considered as final annotation results. Amino acid sequences of the predicted genes were also matched with the corresponding hidden Markov models (HMMs) of AMPs retrieved from the Pfam database^3^ with an *e*-value threshold of 10^–04^ (Mistry et al., 2020). The results of all these analyses were merged as a putative AMP candidates set. In addition, other available sequences of other AMPs and AMP-encoding transcripts derived from Papilionoidea species in public databases, as well as AMPs identified from our next-generation transcriptomic data (GSE142679) of *Papilio clytia* and *Atrophaneura mencius* following lipopolysaccharide (LPS) challenges and verified by Sanger sequencing, were also included for further analyses. The sequences of mature peptides were deduced by alignment with the APD3 database (Wang et al., 2016) and identification of potential cleavage sites in the putative prepropeptides.

### Multiple Sequence Alignment and Phylogenetic Analyses

The deduced butterfly AMPs and homologous sequences available from public databases were subjected to phylogenetic analyses (Supplementary Table 2). Phylogenetic trees were reconstructed using MEGA X (version 10.1.7) software (Kumar et al., 2018), based on peptidyl sequence similarities and differences aligned by ClustalW2 (version 2.1) program^4^ (Larkin et al., 2007, 0) using multiple alignment parameters with default settings.

### Peptide Design

To reveal the evolutionarily conserved positions of the AMPs, entropy calculations were performed, and the residue preference of putative butterfly-derived natural AMPs was investigated and visualized using the WebLogo program^5^ (Crooks et al., 2004). A series of peptide analogs were designed based on the sequence conservation and amino acid frequency at particular positions. Hydropathic characteristics of the amino acids at evolutionarily variable positions were also considered, which retained a consistent hydrophilicity or hydrophobicity to respective positions at the amphipathic α-helices. Estimated physicochemical characteristics of the newly designed AMPs were calculated using PepCalc tool^6^ (Lear and Cobb, 2016). Secondary structures were constructed by homology modeling using the SWISS-MODEL server^7^ (Waterhouse et al., 2018).

### Peptide Synthesis

The designed AMPs were synthesized by the standard Fmoc-based solid-phase peptide synthesis (SPPS) methodology (GL Biochem Ltd., Shanghai, China) on respective Wang resins and Rink Amide MBHA resin for C-terminal amidated peptides. Intramolecular disulfide bond formation was achieved by air-mediated oxidation. Fluorescence-labeled AMPs were synthesized using the same protocol with conjugation of fluorescein isothiocyanate (FITC) and an ε-aminocaproic (Acp) linker at the N-terminus. Synthesized peptides were purified using a high-performance liquid chromatography (HPLC) system (LC3000, Beijing Chuangxin Tongheng, Beijing, China), and the identity of the peptides was confirmed by mass spectrometry (6125B, Agilent Technologies, Santa Clara, CA, United States).

### *In vitro* Antimicrobial Assay

*In vitro* antimicrobial activities of the synthetic peptides against different microorganisms, including the Gram-negative bacteria *Escherichia coli* (ATCC 25922), the Gram-positive bacteria *Staphylococcus aureus* (ATCC 6538P), and the fungus *Candida albicans* (ATCC 10231), were determined by microtiter broth dilution assays (Wiegand et al., 2008). Briefly, the peptides were dissolved and diluted with respective culture mediums to a gradient concentration range of 0.01–32 μmol⋅L^–1^ and were then applied to mid-logarithmic phase microbes with cell densities of approximately 1.0 × 10^8^ CFU⋅ml^–1^ and cultured for 24 h. The absorbance of the cultures was evaluated at 600 nm using a microplate reader (Pulang, China). The minimum inhibitory concentration (MIC) was defined as the lowest concentration of each peptide that exhibited apparent growth suppression on the microbes, while the minimum bactericidal concentration (MBC) was defined as the lowest peptide concentration that resulted in no obvious colony growth of the microorganisms.

### Antibiofilm Assays

Biofilms of the aforementioned microbes were allowed to form in microtiter plates under respective culture conditions in the absence (eradication) or presence (prevention) of AMPs. After incubation, the culture supernatant was discarded, and each well was gently rinsed to remove planktonic bacteria. Biofilms were fixed with methanol and stained with 0.1% crystal violet for 20 min; then the dye bound to the cells was resolubilized with ethanol, and the absorbance of the solution was evaluated at 570 nm using a microplate reader (Abouelhassan et al., 2017). The minimal biofilm inhibitory concentration (MBIC) was defined as the lowest concentration of each peptide that exhibited apparent biofilm inhibition, while the minimal biofilm eradication concentration (MBEC) was defined as the lowest peptide concentration that resulted in biofilm eradication.

### Hemolysis Assay

Hemolysis activity of the synthesized peptides was tested using freshly drawn mouse erythrocytes, which were rinsed three times with phosphate-buffered saline (PBS) and then cultured with peptides at concentrations of 4, 8, 16, 32, and 64 μmol⋅L^–1^ for 2 h (Lin et al., 2013). After centrifugation, 100 μl of the supernatant from each sample was transferred to a microtiter plate, and the cell-free hemoglobin, representing hemolysis, was detected at 550 nm with a microplate reader (Pulang, China).

### Cytotoxicity Assay

To evaluate the potential cytotoxicity on the normal cells, human embryonic kidney (HEK293) cells were used, and the cell viability was assessed by cell counting kit (CCK)-8 assays following the manufacturer’s protocol (Dojindo Inc., Kumamoto, Japan) (Wang et al., 2018). Briefly, the cells were seeded in microtiter plates at a density of 1 × 10^4^ cells per well for 24 h and cultured with increasing concentrations of AMPs ranging from 4 to 64 μmol⋅L^–1^ and cultured for 24 h. The CCK-8 solution was then added to each well, and the cells were incubated for an additional 3 h. The optical density (OD) was measured at 450 nm.

### Membrane Permeability Assay

SYTOX Green uptake assays were performed according to the instructions of the manufacturer (Thermo Fisher Scientific, Waltham, MA, United States) to estimate the membrane permeabilization effect of the AMPs (Jin et al., 2016). Briefly, 1.0 × 10^8^ CFU⋅ml^–1^ microorganisms in logarithmic phase were incubated with AMPs at concentrations of 4× the corresponding MIC value for 1 h. Microbial cells were then collected, washed in Hanks’ balanced salt solution (Solarbio, Beijing, China), and incubated with SYTOX Green Nucleic Acid Stain. The fluorescent intensity, which reflects uptake of SYTOX Green, was measured using a fluorescence microplate reader (Thermo Fisher Scientific), at wavelengths of 485 and 520 nm for excitation and emission, respectively.

### Electron Microscopy

Samples for electron microscopy were prepared according to a previously described method (Hartmann et al., 2010). In general, 1.0 × 10^8^ CFU⋅ml^–1^ exponential growth phase cultures of *E. coli*, *S. aureus*, and *C. albicans* were incubated with AMPs at 4× the corresponding MIC value for 1 h. Microbial cells were then pelleted by centrifugation, fixed with glutaraldehyde solution (Phygene, Fuzhou, China), and stained with osmium tetroxide (OsO_4_). Morphological and ultrastructural changes in the microorganisms were observed and photographed using scanning electron microscopy (SEM; SU8020, Hitachi, Tokyo, Japan) and transmission electron microscopy (TEM; JEM-1200EX, JEOL, Tokyo, Japan).

### Confocal Microscopy

Based on the electron microscopic findings and to further investigate the mechanism of the bacteriostatic activity of AMPs on *C. albicans*, fluorescence-labeled peptides were employed to localize potential targets of the synthetic AMPs. Briefly, *C. albicans* cultures were treated with FITC-labeled AMPs at 4× the corresponding MIC value for 1 h and were then sequentially stained with propidium iodide (PI) and 4′, 6-diamidino-2-phenylindole (DAPI). Images were acquired with a confocal microscope (Ti2-E A1R+, Nikon, Tokyo, Japan).

### Circular Dichroism Assay

Genomic DNA was isolated from *C. albicans* using a fungal genomic DNA extraction kit (Solarbio) following the instructions of the manufacturer. The quantity of the resulting DNA was assessed by NanoDrop 2,000 μ-volume spectrophotometer (Thermo Fisher Scientific). An aliquot of the prepared DNA was then mixed with an equivalent volume of synthetic AMPs at 4× the concentration of respective MIC value. DNA-binding activity of the peptides was detected by measuring the circular dichroism (CD) spectra in quartz cuvettes of 1-mm path length and wavelength ranging from 240 to 330 nm using a Chirascan spectrometer (Applied Photophysics, Leatherhead, United Kingdom) (Garbett et al., 2007).

### *In vivo* Antimicrobial Assay

*In vivo* antimicrobial activities of the designed AMPs were examined in neutropenic mice following subcutaneous inoculation of 100 μl of 1.0 × 10^8^ CFU⋅ml^–1^ *E. coli*, *S. aureus*, or *C. albicans* (Lee et al., 2005). At 12 h post-infection, 100 μl of AMPs at the respective MIC value was applied by subcutaneous injection each day. Mice were sacrificed on day 4, and the infected area was carefully excised and homogenized. The CFU count was determined after overnight incubation of the homogenate aliquots. All animal care and experiments were performed in compliance with institutional and government guidelines and were approved by the Peking Union Medical College Hospital Animal Care and Use Committee.

### Statistical Analysis

All data represent the means ± standard deviations (SDs) of triplicate determinations in three independent experiments. Statistical analyses were performed by *t*-test or one-way analysis of variance (ANOVA) with the least significant difference (LSD) *post hoc* test for multiple comparisons using SPSS Statistics 19.0 software (IBM Corp., Armonk, NY, United States); *p* < 0.05 was considered statistically.

## Results

### *In silico* Screening of Antimicrobial Peptides

Available genomic sequencing datasets from 32 Papilionoidea species of six families (one species from Hesperiidae, one from Lycaenidae, 20 from Nymphalidae, four from Papilionidae, four from Pieridae, and two from Riodinidae) deposited in the GEO database up to January 2019 were downloaded for analyses. From these sequences, 434,811 genes encoding small peptides (<400 residues) were retrieved by Augustus prediction, despite differences in the integrity of the genomic data. Alignment of these sequences with AMPs from RefSeq and Pfam databases resulted in 183 computational translated peptides being annotated as AMPs. Thirty-six of these deduced peptides, including 28 predicted cecropins, five defensins, and three moricins, with high sequence similarities had their presumed propeptide cleavage sites confirmed manually. In addition, two cecropins and one defensin identified from *P. clytia* and *A. mencius* transcriptomic data were also included. This set of peptide sequences was merged with available sequences obtained by database query and literature research (four moricins from *Danaus plexippus*, *Papilio polytes*, *Pieris rapae*, and *Bicyclus anynana*). However, no glycine-rich peptides (such as attacins or gloverins) or proline-rich peptides (such as lebocins) were found in the butterfly genome datasets. Amino acid sequences of the putative AMPs and theoretical physicochemical properties of the deduced mature peptides are displayed in Table 1.

**TABLE 1:** List of antimicrobial peptides of butterflies.

	Source species	AMP sequence	Physicochemical property of mature peptide		
			
			Number of amino acids	MW	PI
**Cecropins**					
1	*Lerema accius*	MKVFNVFLFVFACILALSTVAAAPEP-RWNPFKKLERVGQNIRDGIIKAGPAVAVVGQAASIYKGK	39	4,236.98	11.09
2	*L. accius*	MKVFNVFLFVFACILALSTVAAAPEP-RWNPFKKLEKVGQNIRDGIIKAGPAVAVVGEAANIYKGK	39	4,236.90	10.58
3	*Neruda aoede*	MKFTKVFFFVFACFVALSTVAAAP-WNPFKELEKAGQRVRDAIISAGPAVQVVGQATSIIKGGN	39	4,106.62	10.31
4	*Vanessa tameamea*	MNFAKIFFFIFACIVLTTVSGAPSP-KWKLFKRIEKIGRNVRNGLIKAGPAIQVVGQA	32	3,589.27	12.17
5	*Pieris rapae*	MNFGKLFFFVFACVLALSTVSAAP-KWKIFKKIEHFGQNIRDGLIKAGPALQVVGEAATIYKGK	39	4,354.09	10.47
6	*Calycopis cecrops*	MDFSKILFFIFASLLSLNMVAAAP-WNPLKELERAGQRVRDAIISAGPAVDVVEKTAAIIKGGQQ	40	4,285.84	9.75
7	*Papilio xuthus*	MKYVTIILFVFIAVVAISYVSAEPIP-WNPFKELERAGQNIRDAIISAGPAVDVVARAQKIARGEDVDEDD	44	4,836.22	4.20
8	*L. accius*	MKVFNVFLFVFACILALSTVAAAPEP-RWNPFKKLERVGQNIRDGIIKAGPASRCGGGPSREHIQGKMNLSV	45	4,959.65	11.40
9	*P. machaon*	MHYRNCKTLSGVIGAPEPR-WNPFKKLEKVGQNIRDGIIKAGPAVEVIGQAASIVKPNQGK	41	4,400.07	10.53
10	*P. machaon*	MACLAALSLTTASP-KWKIFKKIEKVGRNVRDGIIKAGPAVAVVGQAATVAK*-G	37	3,946.72	11.23
11	*P. machaon*	MNFAKILFFVVACFAAFSVTSASP-KWKLFKKIEKVGRNIRNGIIKAGPAVQVVGQASQIYKQ*-G	38	4,294.08	11.25
12	*P. machaon*	MKYVTIILFVLVAVIAISYVSAEPIP-WNPFKELERAGQNIRDAIISAGPAVDVVARAQKIARGEDVDEEE	44	4,864.27	4.28
13	*Papilio xuthus*	MNFGKILFFVMACLAALSLTTASP-RWKIFKKIEKVGRNVRDGIIKAGPAVAVVGQAATVVK*-G	37	4,002.78	11.62
14	*Papilio polytes*	MNFAKILFLVVACFAAFSVTSASP-KWKIFKKIERVGQNIRDGIIKAGPAVAVVGQAASIIKPGK	40	4,286.10	11.23
15	*Danaus plexippus*	MNYKRIFFSLLSILLISMVASSPAP-KWKPFKKLEKIGQRVRDGIIKAGPAVQVVGEAAAILKPAQ*-G	40	4,339.16	10.90
16	*D. plexippus*	MNFFRLLFFVALAVMVLSGVSASPSP-RWKFLKKIEKVGRKVRDGVIKAGPAVGVVGQATSIYKGK	39	4,269.07	11.28
17	*D. plexippus*	MDFSKIFFFVFACFLALSNVSAAPSP-KWKIFKKIEKVGRNVRDGIIKAGPAVQVVGQATSIAK*-G	37	4,033.79	11.23
18	*D. plexippus*	MKFGKLLFFVFACIMAFSTVSGAPSP-KWKFFKKIEKVGRNIRDGIIKAGPAVQVLGEAKAIGK	37	4,094.87	10.97
19	*P. rapae*	MNFGELYFLIFACVLALSSVSAAP-KWKIFKKIEHMGQNIRDGLIKAGPAVQVVGQAATIYK*-G	37	4,136.89	10.59
20	*V. tameamea*	MNFAKIFFFIFACIVLTTVSGAPSP-KWKLFKRIEKLGQRVRDGIIKAGPAVGVIGQASTIIK*-G	37	4,074.89	11.62
21	*P. rapae*	MNFGKLFLFVFACVLALSSVSAAP-KWKIFKKIEHMGQNIRDGLIKAGPAVQVVGQAATIYKGK	39	4,323.10	10.69
22	*P. rapae*	MNFGKLFFFVFACVLALSTVSAAP-KWKIFKKIEHMGQNIRDGLIKAGPAVQVVGEAATIYKGK	39	4,324.08	10.47
23	*D. plexippus*	MKFFNLFTFVFACFMVLGLATAAP-WNPFKELEKAGQRVRDAIISAAPAVEVVGQASSILKGKN	39	4,178.73	10.16
24	*Papilio xuthus*	MKYVTIILFVFIAVVAISYVSAEPIP-WNPFKELERAGQNIRDAIISAGPAVDVVARAQKIARGEDVDEDE	44	4,850.25	4.24
25	*Papilio xuthus*	MNFAKILFFVVACFAAFSVTSASP-KWKLFKKIEKVGRNIRNGIIKAGPAVQVVGQASQIYKL*-G	38	4,279.11	11.25
26	*Papilio xuthus*	MNFNKILSFAFVLFAALSSVIAAPEP-RWNPFKKLERVGQNIRDGIIKAGPAVAVVGQAASIIKPGK	40	4,284.00	11.58
27	*Papilio xuthus*	MNFNIILCFIIVFFTSLSGVIGAPEP-KWNPFKKLEKVGQNIRDGIIKAGPAVQVIGQAASIVKPNQGK	42	4,527.26	10.90
28	*Papilio xuthus*	MNFGKILFFVMACLAALSLTTASP-RWKIFKKIEKVGRNVRDGIIKAGPAVAVVEQAATVVK*-G	37	4,074.84	11.14
29	*Papilio clytia*	MNFAKILFFVVACFAAFSVTSASP-RWKLFKKIEKVGRNIRDGIIKAGPAVQVVGQASQIYKL*-G	38	4,308.11	11.16
30	*Atrophaneura mencius*	MNFNRIMSFLFVFFVAICAVSGAPEP-RWNPFKKLEKVGQNIRDGIVKAGPAVGVIGQAASIVKPGK	40	4,227.94	11.16
**Defensins**					
1	*Calycopis cecrops*	MARSYQSMLLLVCISFLVIASAPQNGVAA-DKLIGSC^1^VWGAVNYTSNC^2^NAEC^3^KRRGYKGGHC^4^GSFANVNC^5^WC^6^ET	44	4,797.29	8.02
2	*L. accius*	MVKSYRSVLLLVCVTFLVIVSSPRNEVAA-DKLIGSC^1^VWGAVNYTSDC^2^NAEC^3^KRRGYRGGHC^4^GSFANVNC^5^WC^6^ET	44	4,826.28	7.63
3	*Phoebis sennae*	MVQSYRSMLLLVCVSFLVIVSSPSNSAAA-DKLIGSC^1^VWGAVNYTSDC^2^NKEC^3^KRRGYKGGHC^4^GSFANVNC^5^WC^6^ET	44	4,855.36	8.01
4	*Papilio polytes*	MKGRVIIFVVLIGLAVVAAAEVEEYNESSLVTRLKRETIMVKPPK-GC^1^VFYEC^2^IARC^3^RQRGYLSGGYC^4^TINGC^5^QC^6^L*-G	30	3,330.83	8.01
5	*Papilio memnon*	MKGRVIIFVILIGLAVAAAAEVEQSKESSLVSRLKRETIMMKLPK-GC^1^VFYEC^2^IARC^3^RQRGYLSGGYC^4^TINGC^5^QC^6^L*-G	30	3,330.83	8.01
6	*P. clytia*	MKGHVIFFFVFLIGLTYVTSVALKDIRHPSFLTRLKRETIMVKPPK-GC^1^IFYEC^2^IARC^3^RQRGHLSGGYC^4^TINGC^5^QC^6^L*-G	30	3,318.82	8.02
**Moricins**					
1	*D. plexippus*	MKLIAVLLVMLCLMSVFDTLEASP-ARIPIGAIRKGAKAVGKGLRAINIAGTVHDIVEVFKPRKRKH	42	4,543.4	12.26
2	*Calycopis cecrops*	MKIYGLFLIVISILALLVAPNEAKP-GKIPIGAIKKGAKLVGKGLKALNIASTANDVYHFFHHKRKH	41	4,465.24	11.01
3	*Calycopis cecrops*	MKFFGLFLVVLSLLALLASPSEARP-GKIPIGAIKKGAEVVGKGLKALNIASTANDVYKFFHHKKKH	41	4,415.17	10.65
4	*D. plexippus*	MKFFSVFVVIVTVLAVFLGTGEARP-GKIPINAIRKGAKAVGHGLRALNIASTAHDIVSAFKHKKRKH	42	4,511.27	12.19
5	*Papilio polytes*	MDFTKMFVLLFGILAIFMGRCNAKP-GIPIGAIKKGGQWIRKGFGVLSAAGTAHEVYSHVKNRRN	39	4,174.75	11.55
6	*P. rapae*	MDFRKIFLLIVLSVFAIFGSEARP-GKIPKAVIKKGAKLVGNGLKALNVASTVHDIYSALHHKKKKH	42	4,499.34	10.90
7	*Bicyclus anynana*	MKFTSLLILILGVFSLFIGASDARP-KIPINAIRKGARAVGKGLRMINYASTAHDIASMFHKKKRKH	41	4,615.47	12.04

Amino acid sequences of all deduced butterfly antimicrobial peptides are shown above, with larger font size representing mature peptides. Asterisk (*) refers to removal of glycine and subsequent amidation of the penultimate residue at the C-terminus. Superscript numerals (1–6) in defensin indicate the order of cysteine residues that form three pairs (1–4, 2–5, and 3–6) of disulfide bridges. AMP, antimicrobial peptide; MW, molecular weight; PI, isoelectric point.

#### Cecropins

**FIGURE 1:**
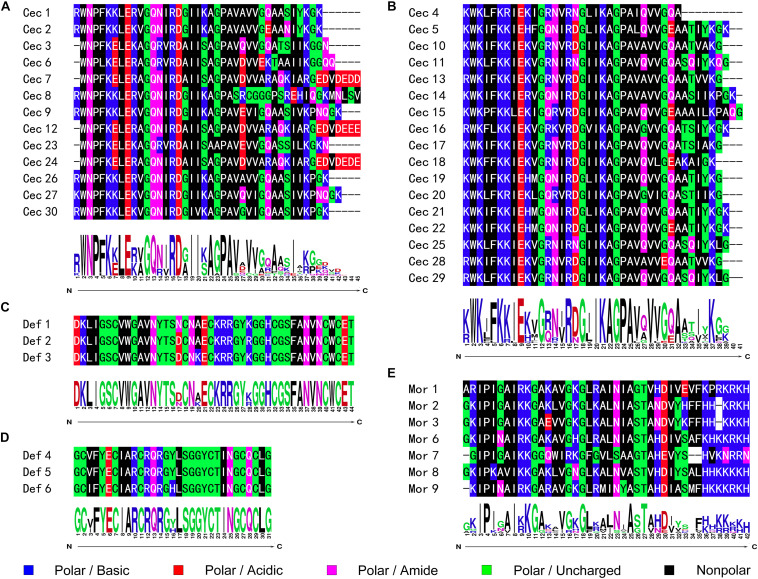
Sequence alignment and logo analysis of naturally occurring AMPs from butterflies. The sequence differences therein suggested that both cecropins and defensins could be further subclassed into two categories, respectively. The peptidyl sequence similarities and site-specific preference of **(A)** cecropins subclass A; **(B)** cecropins subclass B; **(C)** defensins subclass A; **(D)** defensins subclass B; and **(E)** moricins were analyzed and displayed as graphics.

**FIGURE 2:**
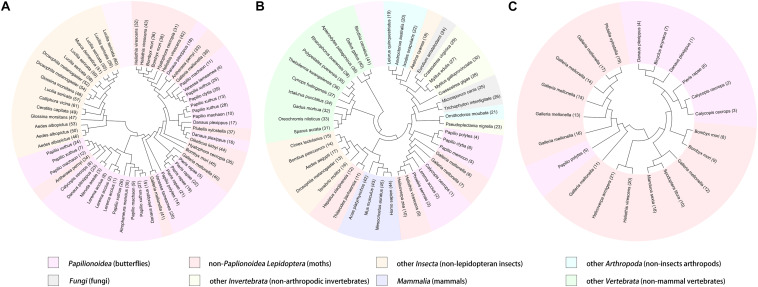
Phylogenetic classification and visualization of butterfly AMPs. The analysis was conducted based on the amino acid sequences of cecropins **(A)**, defensins **(B)**, and moricins **(C)**, among butterfly AMPs and their homologous sequences.

As the first discovered and one of the most intensely studied classes of AMPs, cecropins are characterized by their cationic-residue-rich and alpha-helical structures, and their potent and wide-spectrum antimicrobial activities against different microbes (Bulet et al., 2004). Cecropins consist of a signal peptide and propeptide at the N-terminus of the mature peptide, and most cecropins also contain tryptophan (Trp) at the first or second position and a carboxamide modification at the N-terminus of the mature peptide (Yi et al., 2014). Based on the bioinformatics analysis and data from previous studies, 30 cecropins were identified from different Papilionoidea species. These cecropins had an average length of 39.23 ± 2.67 amino acids and an average molecular weight (MW) of 4,290.86 ± 289.78 g⋅mol^–1^. All deduced sequences were presumed to possess a Trp residue at the N_1_ or N_2_ position, while one-third of the sequences were predicted to have C-terminal amidation by the presence of a glycine (Gly) residue at the C_1_ position. A tetrapeptide motif AGPA that formed the hinge region between the two α-helices featured consistently in almost all cecropins. According to the alignment results, the identified cecropins could be subdivided into two categories that contained prominent sequence differences around the Trp residue (Figures 1A,B). Phylogenetic classification further suggested that these two subclasses of cecropins had diphyletic origin, although the result also illustrated that the two clusters of peptides and other lepidopteran cecropins could be derived from a single ancestral sequence (Figure 2A).

#### Defensins

Defensins represent a class of evolutionarily ancient AMPs that are present in nearly all multicellular organisms and play important roles in humoral defense reactions (Machado and Ottolini, 2015). The predominant characteristic of defensins is the highly conserved motif of cysteine (Cys) residues, through which intracellular disulfide bridges are formed to maintain the secondary structure despite differences in the localization of the Cys residues and the pairing patterns thereof ^[14]^. In contrast to cecropins, the overall sequence similarity of the defensins was not apparent, and the phylogenetic analysis revealed complex relationships among diverse organisms (Figure 2B). However, the amino acid sequences of the deduced defensins in the Papilionoidea superfamily were highly conserved. For example, three transcripts from *Calycopis cecrops* were predicted to differ by nucleotide sequences and corresponding precursor peptides but shared the same mature peptide sequences, which were also identical to those of a defensin from *Archaeoprepona demophon* (Landon et al., 2004). This suggested that certain sequences might be preferable to the host defense system of butterflies. After removal of duplicated sequences, six unique defensins were identified from the genomic and transcriptomic data (Table 1) and could be categorized into two subgroups according to the alignment results and theoretical physicochemical parameters (Figures 1C,D). Sequences of the first cluster of butterfly defensins contained 44 residues and had an average MW of 4,826.31 ± 29.04 g⋅mol^–1^, while sequences of the other subgroup were composed of 30 amino acids and had a mean MW of 3,326.83 ± 6.93 g⋅mol^–1^. The Cys motifs in the two subgroups were C-X_10_-C-X_3_-C-X_9_-C-X_7_-C-X_1_-C and C-X_4_-C-X_3_-C-X_10_-C-X_4_-C-X_1_-C, which were within the range of reported Cys patterns of invertebrate defensins (Seufi et al., 2011). The distinct Cys motifs and primary sequences of the two subgroups of butterfly defensins also reflected the far molecular distance in the phylogenetic analysis, whereas the high sequence similarity within each respective cluster indicated that a tight phylogenetic relationship exists between defensins in the same subgroup (Figure 2B).

#### Moricins

Moricins are a class of highly positively charged AMPs that are broadly effective against diverse microorganisms and feature a long α-helix secondary structure and a cluster of basic amino acid residues in the C-terminal region (Hemmi et al., 2002). Moricin peptides, initially isolated from the hemolymph of *Bombyx mori* larva under bacterial challenge and later discovered from several other moth species, are considered to only be expressed in members of the order Lepidoptera (Xu et al., 2019). Although limited moricin sequences of butterfly origin have been reported, the current study identified a total of seven moricins by genomic analysis and database retrieval. These moricin sequences had an average amino acid length of 41.14 ± 1.07, an MW of 4,460.66 ± 140.66 g⋅mol^–1^, and a calculated mean isoelectric point of 11.51 ± 0.67, which was greater than that of the cecropins (10.32 ± 2.12) and defensins (7.95 ± 0.16). Despite the limited number of published moricin sequences, the multiple sequence alignment and phylogenetic analyses revealed significant sequence similarity and a monophyletic origin among all naturally occurring moricins (Figure 2C). The amphipathic segment of the α-helix at the N-terminus of the moricins is critical for the antibacterial activities of these peptides, but the function of the positively charged segment at the C-terminus remains unknown (Hemmi et al., 2002; Oizumi et al., 2005). However, amino acid frequency analysis at the relative region in the current study suggested that net positive charge rather than site-specific preference could control the activities of moricins (Figure 1E).

### Design of Butterfly Antimicrobial Peptide Analogs

**FIGURE 3:**
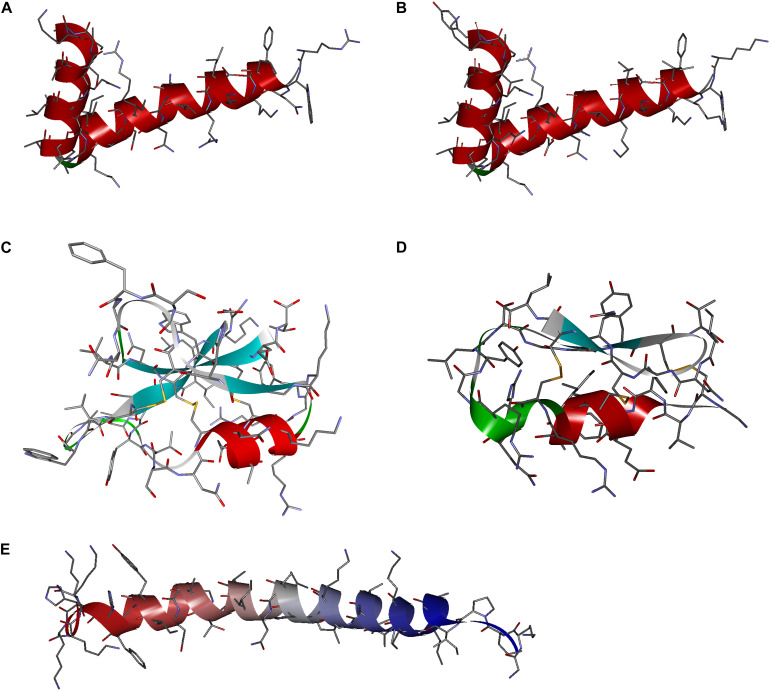
Secondary structures of the designed peptide. The three-dimensional shapes of the synthetic molecules, e.g., **(A)** cecropin A, **(B)** cecropin B, **(C)** denfensin A, **(D)** denfensin B, and **(E)** moricin A, were constructed by homology modeling, which demonstrated typical structural features of respective classes of antimicrobial peptides (AMPs).

Analysis of the sequence conservation and amino acid frequency at particular positions facilitated the design of a series of peptides with antimicrobial potential, including two cecropins [cecropin A (CecA) and cecropin B (CecB)], two defensins [defensin A (DefA) and defensin B (DefB)], and one moricin [moricin A (MorA)], corresponding to aforementioned butterfly AMP classifications. The amino acid length, theoretical MW, and isoelectric point of all designed peptides were within the range of corresponding parameters analyzed (Table 2 and Supplementary Table 3). The homology modeling results displayed classical secondary structural characteristics of cecropins, defensins, and moricins, which were in accordance with the corresponding conservative structural features described above (Figure 3). Following this *in silico* analysis, the peptides were chemically synthesized for further evaluation (Supplementary Figure 2).

**TABLE 2:** Amino acid sequences and physicochemical properties of the designed peptides.

AMPs	Family	Sequence	Physicochemical property		
			
			Number of residues	MW	PI
CecA	Cecropin	RWNPFKKLERVGQNIRDGIIKAGPAVAVVGQAASIAK-NH_2_	37	3,958.62	11.93
CecB	Cecropin	KWKIFKKIEKVGRNIRDGIIKAGPAVQVVGQAATIYKGK	39	4,310.14	11.12
DefA	Defensin	DKLIGSCVWGAVNYTSDCNAECKRRGYKGGHCGSFANVNCWCET	44	4,804.36	7.45
DefB	Defensin	GCVFYECIARCRQRGYLSGGYCTINGCQCL-NH_2_	30	3,336.91	8.12
MorA	Moricin	GKIPIGAIKKGAKAVGKGLKALNIASTAHDIYSFHHKKKKH	41	4,363.17	11.06

*AMP, antimicrobial peptide; MW, molecular weight; PI, isoelectric point.*

### *In vitro* Antimicrobial Assay

*In vitro* antimicrobial activities of the peptides were examined by determination of the MIC and MBC values against representative microorganisms (*E. coli*, *S. aureus*, and *C. albicans*) of public concern using microtiter broth dilution assays. Despite different biological effects among the AMPs, the synthesized cecropins, defensins, and moricins all exhibited potent bactericidal actives against both Gram-positive and Gram-negative bacteria, with MIC and MBC values in micromolar and sub-micromolar concentration ranges; cecropins were the most effective agents. In addition, while cecropins exhibited modest antifungal effects, the synthetic moricin possessed relatively strong fungicidal activities, with low micromolar MIC and MBC values (Table 3). No apparent hemolysis was detected at concentrations exceeding the respective MIC and MBC values (Supplementary Figure 3).

**TABLE 3:** *In vitro* antimicrobial activities of the designed peptides.

AMPs	*Escherichia coli*		*Staphylococcus aureus*		*Candida albicans*	
			
	MIC	MBC	MIC	MBC	MIC	MBC
CecA	0.5	2	1	4	16	32
CecB	0.5	1	1	2	8	16
DefA	8	32	1	16	*ND*	*ND*
DefB	2	16	0.5	16	*ND*	*ND*
MorA	1	2	2	4	1	2
Cec(Ctrl)	0.5	1	1	2	16	32
Def(Ctrl)	1	32	8	32	*ND*	*ND*
Mor(Ctrl)	1	2	2	4	2	4

**Hyalophora cecropia* cecropin A [Cec(Ctrl)], *Galleria mellonella* defensin [Def(Ctrl)], and *Manduca sexta* moricin [Mor(Ctrl)] served as respective controls. The results of antimicrobial activities evaluation are presented as minimal inhibitory concentration (MIC) and minimum bactericidal concentration (MBC) value (μmol⋅L^–1^) against different microorganisms, while *ND* stands for not detected.*

*AMP, antimicrobial peptide.*

### Antibiofilm Assay

The biofilm inhibitive and degradative effects of the synthetic peptides were quantitated by measuring the MBIC and MBEC values against *E. coli*, *S. aureus*, and *C. albicans*, which represent Gram-negative bacteria, Gram-positive bacteria, and fungi, respectively. All the synthetic peptides prevented the formation of biofilms of both Gram-negative and Gram-positive bacteria and eliminated pre-formed biofilms of both bacterial species. Congruent with the results of the antifungal assays, neither of the defensins displayed visible antibiofilm activity against *C. albicans*. In contrast, MorA displayed the greatest antibiofilm activities among all the designed AMPs (Table 4).

**TABLE 4:** Antibiofilm activities of the designed peptides.

AMPs	*Escherichia coli*		*Staphylococcus aureus*		*Candida albicans*	
			
	MBIC	MBEC	MBIC	MBEC	MBIC	MBEC
CecA	2	8	2	16	16	32
CecB	2	8	2	16	16	32
DefA	8	32	4	16	*ND*	*ND*
DefB	8	32	2	16	*ND*	*ND*
MorA	4	8	4	16	4	16
Cec(Ctrl)	2	8	2	16	16	32
Def(Ctrl)	8	32	4	16	*ND*	*ND*
Mor(Ctrl)	4	8	4	16	4	16

**Hyalophora cecropia* cecropin A [Cec(Ctrl)], *Galleria mellonella* defensin [Def(Ctrl)], and *Manduca sexta* moricin [Mor(Ctrl)] served as respective controls. The results of antibiofilm activities evaluation are presented as the minimal biofilm inhibition concentration (MBIC) and the minimal biofilm eradicating concentration (MBEC) value (μmol⋅L^–1^) against different microorganisms, while *ND* stands for not detected.*

*AMP, antimicrobial peptide.*

### Membrane Permeabilization Assay

**FIGURE 4:**
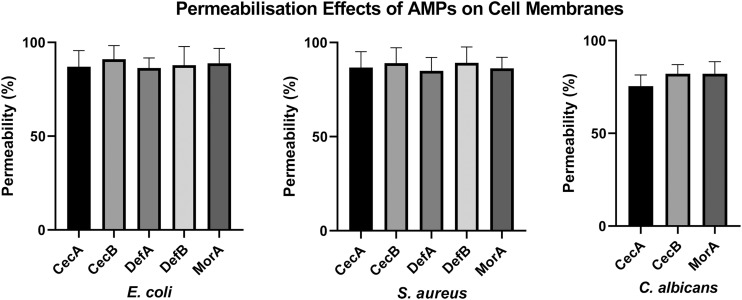
Evaluation of membrane destruction effects of antimicrobial peptides (AMPs) by SYTOX Green uptake assay. Results demonstrated the synthetic peptides induced significant membrane permeability changes against different microorganisms.

**FIGURE 5:**
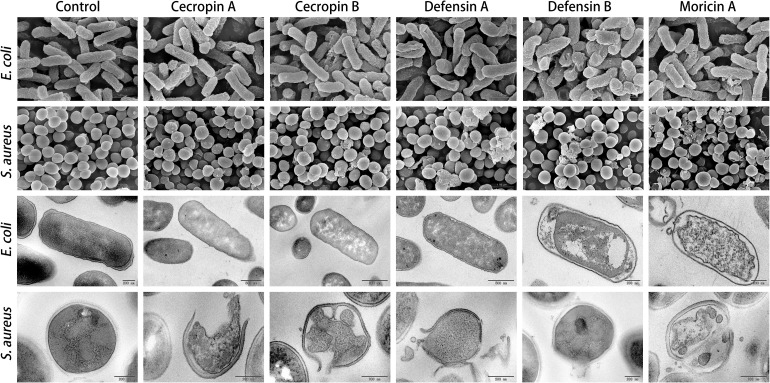
Electron microscopic analysis of bacteria treated with synthetic antimicrobial peptides (AMPs). Representative images of both scanning electron microscopy (SEM; upper panels) and transmission electron microscopy (TEM; lower panels) showed significant changes on cell morphology induced by AMPs at 4× of corresponding minimum inhibitory concentration (MIC) value, compared with the intact smooth surfaces of the untreated control group (left panel). The TEM assay further displayed marked decreases in cell densities that resulted from peptide intervention. These findings suggest membrane permeabilization mechanism being involved in bactericidal activities of the synthesized butterfly AMPs.

**FIGURE 6:**
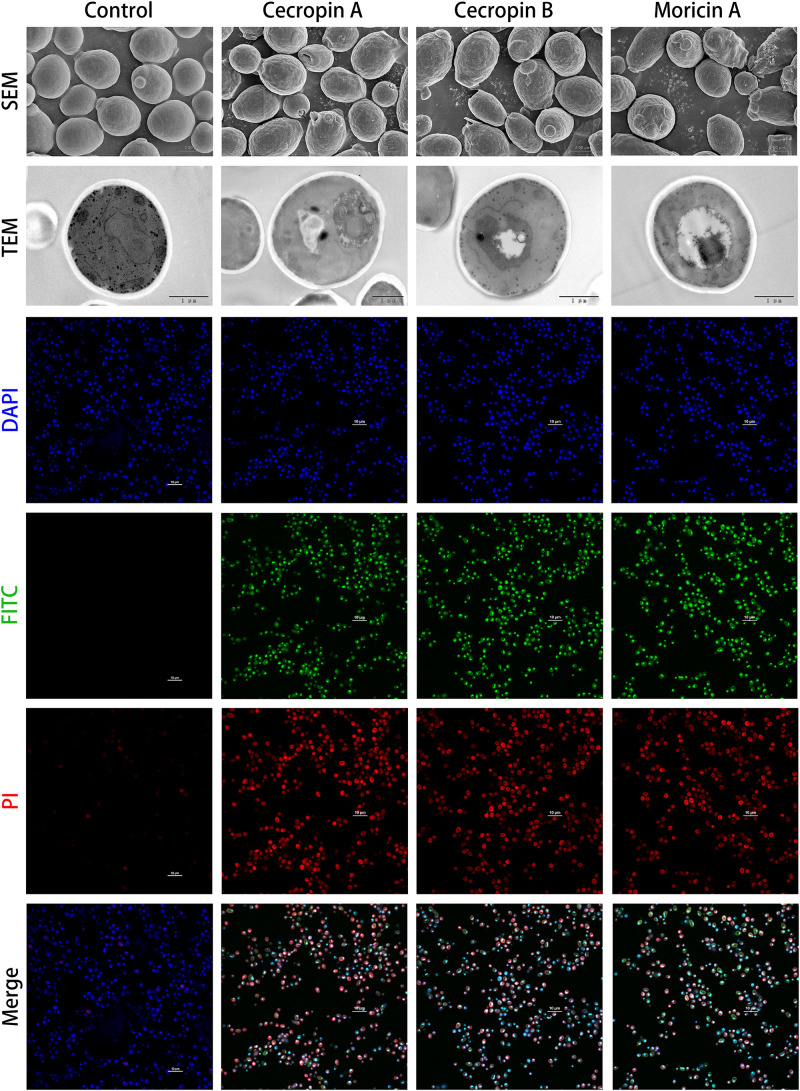
Microscopic investigation of antifungal capabilities of designed antimicrobial peptides (AMPs). The antifungal effects of cecropins and moricins, which had been confirmed by microtiter broth dilution assays (Table 3), were analyzed by combination of scanning electron microscopy (SEM; first upper panel), transmission electron microscopy (TEM; second upper panel), and confocal fluorescence microscopies (lower panels). Although there were no apparent morphological changes observed by electron microscopies, the confocal microscopy demonstrated the fluorescein isothiocyanate (FITC) (green) labeled peptides colocalized with DAPI (blue)-stained fungus nucleus, and the FITC fluorophore showed high spatial overlaps with propidium iodide (PI) (red) fluorescence, which indicates a probable cell death induction mechanism caused by DNA binding.

The membrane disruption effects of the synthesized peptides on the representative microorganisms were analyzed using a SYTOX Green uptake assay as well as SEM and TEM. SYTOX Green, a membrane integrity stain, is unable to enter intact cell membranes; hence, the fluorescence intensity from this stain is positively related to membrane permeability. SYTOX Green staining indicated significant alteration of cell membrane permeability in the assayed microorganisms exposed to the synthetic AMPs (Figure 4). Observations of both Gram-negative and Gram-positive bacteria by electron microscopy revealed distinct morphological and biophysical changes induced by all of the synthetic AMPs (Figure 5). These observations were indicative of a typical mechanism of AMPs binding to and disrupting the cell membranes, resulting in leakage of cell contents. However, images of *C. albicans* showed only a slightly changed cell morphology and a relatively dense internal structure, which indicated that an alternative mechanism might be involved in the antifungal activities of the synthetic AMPs (Figure 6).

### *In vitro* Peptide Localization Assay

To further elucidate the underlying antifungal mechanism of the designed AMPs, FITC-labeled peptides were synthesized and used for localization of the potential binding targets of the synthetic AMPs by confocal fluorescence microscopy. The synthetic cecropins and moricin targeted the DAPI-stained nucleus of *C. albicans* (Figure 6). In addition, the FITC fluorescence showed strong colocalization with the PI signal. DNA-binding activities of the synthetic AMPs were verified by CD spectroscopy, which indicated detectable DNA structural changes (Supplementary Figure 4). Therefore, it was hypothesized that the cationic AMPs exert their antifungal activities by binding to the anionic nucleic acids, thus interfering with bioprocesses and resulting in cell death.

### *In vivo* Antimicrobial Assay

**FIGURE 7:**
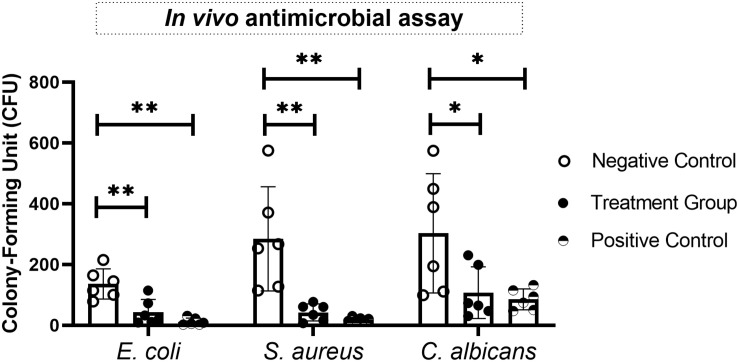
Evaluation of *in vivo* antimicrobial effects of synthetic antimicrobial peptides (AMPs) on various bacteria and fungi in a skin infection mice model. The most effective antibacterial (CecB against *Escherichia coli* and *Staphylococcus aureus*) and antifungal (MorA against *Candida albicans*) agents in the *in vitro* assays were examined in this study, and the antimicrobial activities were expressed as CFU per skin sample. Statistical analyses were performed by *t*-test, and asterisks indicate significant difference (^∗^*p* ≤ 0.05, ^∗∗^*p* ≤ 0.01).

Finally, the *in vivo* antimicrobial activities of the most promising AMPs against bacteria (CecB) and fungi (MorA) were evaluated in a skin infection mouse model. CFU counts for the bacteria and fungus in the presence of the tested AMPs were significantly less than those of the respective controls. This demonstrated that the synthetic cecropin could attenuate growth of both *E. coli* and *S. aureus* in the animal model, while the synthetic moricin could suppress proliferation of *C. albicans in vivo* (Figure 7). These findings suggested that CecB and MorA may have therapeutic potential under different infectious circumstances.

## Discussion

Numerous AMPs have been identified from natural resources, and many more have been synthetically created by modification and *ab initio* design based on sequence and structural features of the naturally occurring molecules (Hale and Hancock, 2007). Many AMPs are currently in preclinical or clinical development for the treatment of various infectious diseases, and most of these AMPs were of natural origin (Greber and Dawgul, 2017). Conventionally, naturally occurring AMPs are predominantly identified by cDNA cloning and sequencing and/or *de novo* peptide sequencing. Moreover, by taking advantage of precursor sequence conservation of known AMPs within *Pisces* and *Amphibia* (fishes and amphibians), a more productive shotgun cloning approach and strategies for database retrieval have been developed to facilitate the identification of homologous sequences (Wang et al., 2012; Liu et al., 2015). However, precursor sequence-based methodologies are not applicable for screening butterfly AMPs, since such sequence conservation has not been revealed among butterfly species. Nevertheless, dozens of naturally occurring AMPs have been recognized from various lepidopteran insects (moths and butterflies) insects, although only a few are derived from Papilionoidea species (butterflies). Butterfly species could therefore be a hidden treasure for natural AMP discoveries and may provide potential templates for peptide design. The availability of an increasing number of high-throughput sequencing datasets from butterflies facilitated the design of an integrated bioinformatics analysis method for the efficient discovery of naturally occurring AMPs.

To process large-scale genome data, many computational tools have been developed that can perform sequence assembly and gene prediction and identification. In the context of the current study and identifying AMPs, protein-encoding portions of transcripts are deducible by ORF analysis, which can be accomplished by using software such as ORFfinder. Next, the signal peptide of propeptides can be predicted by calculation of the potential cleavage sites, which is achieved by using a program such as SignalP. Furthermore, despite the lack of software available to analyze other post-translational modifications, the mature peptide can still be identified by homologous sequence alignment and prediction of cleavage sites (Wang and Hu, 2017). In general, the N-terminus of a pro-AMP undergoes a two-step proteolytic cleavage by signal peptidase and dipeptidyl aminopeptidase (Boman et al., 1989), while peptides with a Gly residue present at the C-terminus are subjected to an amide modification by a two-step sequential catalytic reaction with peptidylglycine α-amidating monooxygenase (PAM) (Kim and Seong, 2001). Based on this bioinformatics analysis method, a total of 36 new, naturally occurring AMPs of butterfly origin were identified from the published genomic data used in the current study. This was substantially greater than the number of previously reported, naturally occurring butterfly AMPs. In addition, the designed peptides, which acted as representatives of the discovered natural AMPs, exhibited potent and wide-spectrum antimicrobial effects with typical membrane-disruption activities. Therefore, the genome-wide *in silico* screening is suggested to be an effective and practical method for identification of naturally occurring AMPs.

The findings from this study add to previous discoveries, and it is our understanding that three classes of AMPs have been found from Papilionoidea species; these classes are cecropins, defensins, and moricins. Of the three classes, cecropins are predominantly expressed by lepidopterans and dipterans and are primarily responsible for the host defense of lepidopterans. Although α-helical AMPs nominated as cecropins have been identified from other species, the sequences of these molecules exhibit a substantially lower degree of similarity to insect cecropins. Defensins have rarely been discovered from moths or butterflies but are ubiquitously expressed throughout multicellular organisms. In contrast, moricins have been identified only in Lepidoptera species. Another class of AMPs unique to the order Lepidoptera is the lebocins; however, no butterfly lebocin sequences were found in the current study. Phylogenetic analyses revealed a monophyletic origin within butterfly cecropins and moricins, which is yet another demonstration of the monophyletic origin of the superfamily Papilionoidea. The molecular distances among defensins of butterfly origin were relatively far, which was expected since it has been proposed that higher variabilities of insect defensins exist among closely related species instead of among distantly related insects (Bulet et al., 2004).

The molecular design of the synthetic peptides in this study was generally based on the mimicry of natural AMPs. The pronounced sequence and structural features, e.g., α-helical structures, C-terminal amidation, disulfide bridges, and the positively charged C-terminal segment, were included in the synthetic peptide design (Table 1 and Figure 3). However, due to the possibility of producing the designed AMPs by microorganism expression systems in the future, more complex chemical modification was not considered in this research. It is recognized that the positively charged amphipathic α-helix of cecropins plays a dominant role in their antimicrobial activities, allowing them to partition into the phospholipid bilayers and disrupt microbial membranes (Hancock and Rozek, 2002). The C-terminal hydrophobic helix of cecropins is believed to have a synergistic effect and provide selectivity against Gram-negative bacteria (Lee et al., 2015). Moricins, which possess an N-terminal amphipathic segment in the one large α-helix structure, were presumed to share a similar mechanism as that of the cecropins. The amphipathicity of the α-helices of the designed peptides was visualized *via* helical wheel projection (Supplementary Figure 5). Furthermore, C-terminal amidation is suggested to be a common modification of the cecropins and is presumed to contribute to the broad-spectrum antimicrobial activity of these peptides (Yi et al., 2014). Among the previously discovered butterfly cecropins, papiliocin from *Papilio xuthus* (Kim et al., 2010) and DAN1 and DAN2 from *D. plexippus* (Duwadi et al., 2018) were proposed to have amidated C-termini, although a non-Gly amino acid as the ultimate residue in DAN2 indicated a natural, non-amidated terminus. On the contrary, hinnavin I and hinnavin II (Yoe et al., 2006) from *P. rapae* were reported to have carboxyl C-termini, even if the presence of a Gly residue at the C-terminus of hinnavin II suggested a potential amidated modification. There were no apparent C-terminal amidation tendencies within the naturally occurring butterfly cecropins examined in the current study (Table 1). Moreover, antimicrobial activities of the amidated analog of CecB were not augmented when compared with the natural prototype, which suggested that C-terminal amidation may not be required for cecropins derived from butterflies. A C-terminal amidation modification has also been observed in a group of natural butterfly defensins, and this modification was relatively rare among other species (Bruhn et al., 2009). C-terminal amidation could be postulated to neutralize the negative charge and increase resistance to exopeptidase hydrolyzation. Further studies are required to elucidate the underlying functions of C-terminal amidation of AMPs. A more conserved character of the defensins is the Cys-stabilized αβ (CSαβ) motif signature, which is considered as the main functional structural basis, despite certain mammalian defensins lacking α-helices (de Oliveira Dias and Franco, 2015). Moreover, the positively charged segment at the C-termini of moricins is the most distinct feature distinguishing them from other α-helical AMPs. Although tetra- and penta-peptide motifs at the positively charged part of moricin have been proposed in other species (Hemmi et al., 2002; Oizumi et al., 2005), the butterfly moricin sequences showed diverse residual propensities in this region. Combined with the confocal microscopy and CD spectroscopy results, which demonstrated that moricins could penetrate the cell membrane and bind to the cell nucleus (Figure 6 and Supplementary Figure 3), it could be arguably hypothesized that the basic amino acid cluster significantly increased the net charge of moricins and hence facilitated the interaction with the negatively charged nucleic acids.

AMPs are active against a broad range of microorganisms, but selectivity is also evident toward respective microbes. The activity of cecropins is thought to be particularly directed against Gram-negative bacteria, while that of defensins and moricins is mainly targeted toward Gram-positive bacteria (Hara and Yamakawa, 1995). However, the *in vitro* antimicrobial assay in the current study revealed that all designed peptides, particularly cecropins, exerted potent but generally undifferentiated activities against Gram-negative and Gram-positive bacteria, with cecropins and moricin exhibiting slightly higher antimicrobial preference against Gram-negative bacteria and defensins showing a stronger effect on Gram-positive bacteria (Table 3). It was not surprising that the designed moricin exhibited predominant antimicrobial activity against Gram-negative bacteria, since the high amphipathicity and net charge of this class of AMP are thought to be positively associated with the activities toward Gram-negative bacteria (Wang et al., 2017). In accordance with this, both SEM and TEM illustrated that the designed peptides in the current study could exert their bactericidal activities through a mechanism of membrane binding and disruption. Furthermore, the synthetic cecropins and moricin, especially the latter, also demonstrated antifungal potential through a possible DNA-binding mechanism. The *in vivo* experiment provided further evidence that the designed peptide CecB had important therapeutic functions in bacterial elimination, while also showing that the synthetic MorA could inhibit fungal growth to some extent (Figure 7). These preliminary findings suggest that the synthetic AMPs, primarily CecB and MorA, are promising antimicrobial agents that warrant further investigation. All the synthetic sequences generated in the current study demonstrated potent antimicrobial activities, which further supports the rationality and necessity of selecting sequences and structures that have been phylogenetically conserved through evolution. The characteristics of natural butterfly AMPs could be explored further through more extensive sequence and structural optimization as well as considering the possibility of peptide expression. Furthermore, the lower antimicrobial activity of defensins does not mean that these AMPs are less important; there is accumulating evidence for the existence of immunoregulatory properties of defensins on host immunity and resultant therapeutic potential, as well as the occurrence of functional synergies between a defensin and other AMPs or antimicrobial agents (Pazgier et al., 2007; Lazzaro et al., 2020). Examination of these theories was beyond the scope of the current study.

The limited numbers of representative microorganisms applied in the current study mean the antimicrobial spectrum and bioactivities of the AMPs require further evaluation to obtain a more detailed characterization of these molecules. For example, AMPs have been found that can target and lyse phosphatidylserine (PS), thereby exposing negatively charged tumor cells, and these AMPs are even active against drug-resistant tumor cell variants and may not affect their healthy counterparts (Riedl et al., 2011). In addition, some AMPs were reported to inhibit and degrade biofilm formation by coating the bacterium or the biomaterial surface (Segev-Zarko et al., 2015); however, the mechanism of antibiofilm activity was not investigated in this study. AMPs have also demonstrated antiviral effects on different viruses, ranging from influenza to human immunodeficiency virus (HIV), through multiple mechanisms (Vilas Boas et al., 2019). Antiviral effects of the synthetic AMPs were not explored in the current study. Despite these limitations, the current study provides valuable information on butterfly AMPs and an efficient and practical methodology for AMP screening and design.

## Conclusion

This study presented an overview of the landscape of butterfly AMPs and insights into their diverse sequence and structural features. Based on these sequences, a series of potent and wide-spectrum peptides with respective antimicrobial mechanisms were successfully designed. This study not only provided a practical strategy for high-throughput natural AMP discoveries but also produced a rational methodology for AMP design, and this will be of importance for future studies.

## Data Availability Statement

The datasets presented in this study can be found in online repositories. The names of the repository/repositories and accession number(s) can be found in the article/Supplementary Material.

## Ethics Statement

The animal study was reviewed and approved by Peking Union Medical College Hospital Animal Care and Use Committee, Peking Union Medical College Hospital.

## Author Contributions

MW and XH conceived the study. WZ, SL, and XH performed the *in silico* experiment. MW and ZZ carried out the wet lab experiment. MW prepared the draft manuscript. XH reviewed the draft manuscript. All authors read and approved the final manuscript.

## Conflict of Interest

The authors declare that the research was conducted in the absence of any commercial or financial relationships that could be construed as a potential conflict of interest.

## Publisher’s Note

All claims expressed in this article are solely those of the authors and do not necessarily represent those of their affiliated organizations, or those of the publisher, the editors and the reviewers. Any product that may be evaluated in this article, or claim that may be made by its manufacturer, is not guaranteed or endorsed by the publisher.

## Footnotes

1
http://augustus.gobics.de/

2
http://www.ncbi.nlm.nih.gov/RefSeq/

3
http://pfam.xfam.org/

4
http://www.ebi.ac.uk/tools/clustalw2

5
http://weblogo.berkeley.edu/

6
http://pepcalc.com

7
http://swissmodel.expasy.org

## Supplementary Material

Supplementary Figure 1 Schematic illustration of the experimental design.

Supplementary Figure 2 HPLC profiles and spectrometric data of the synthetic AMPs.

Supplementary Figure 3 Evaluation of hemolytic and cytotoxic activities of synthetic AMPs. The hemolysis effects (left) and cytotoxicity on normal cells (right) of the synthetic peptides at different concentration were tested on mice erythrocytes and HEK293 cells, respectively. PBS and Triton-X100 were used as the negative and positive control. No apparent hemolysis or cytotoxicity was detected among groups.

Supplementary Figure 4 CD spectrum of the designed AMPs interacted with fungal genomic DNA. The top curve corresponded to free DNA, while the lower curves were that mixed with respective AMPs. The decreased intensities of genomic DNA molar ellipticity exhibited occurrence conformational alteration of DNA that induced by AMPs, which suggested a DNA-binding mechanism of the designed sequences.

Supplementary Figure 5 Helical wheel projection analysis of the designed peptide. **(A)** N-terminal amphiphilic helix of cecropin A; **(B)** C-terminal hydrophobic helix of cecropin A; **(C)** N-terminal amphiphilic helix of cecropin B; **(D)** C-terminal hydrophobic helix of cecropin B; **(E)** helical segment of defensin A; **(F)** helical segment of defensin B; **(G)** helical segment of moricin A.

Supplementary Table 1 Papilionoidea species and their high-throughput sequencing data used in this study.

Supplementary Table 2 The AMP sequences for phylogenetic analysis in this study.

Supplementary Table 3 Sequence alignment of the synthetic and naturally occurring AMPs.
