# Supplementary material for: Identification and Characterization of Antimicrobial Peptides From Butterflies: An Integrated Bioinformatics and Experimental Study
Source: Front Microbiol. 2021 Aug 26;12:720381. doi: 10.3389/fmicb.2021.720381 (PMC8427292; doi:10.3389/fmicb.2021.720381)
Supplement: Supplementary Figure 1 — Schematic illustration of the experimental design. [file Data_Sheet_1.docx]

Supplemental material

**
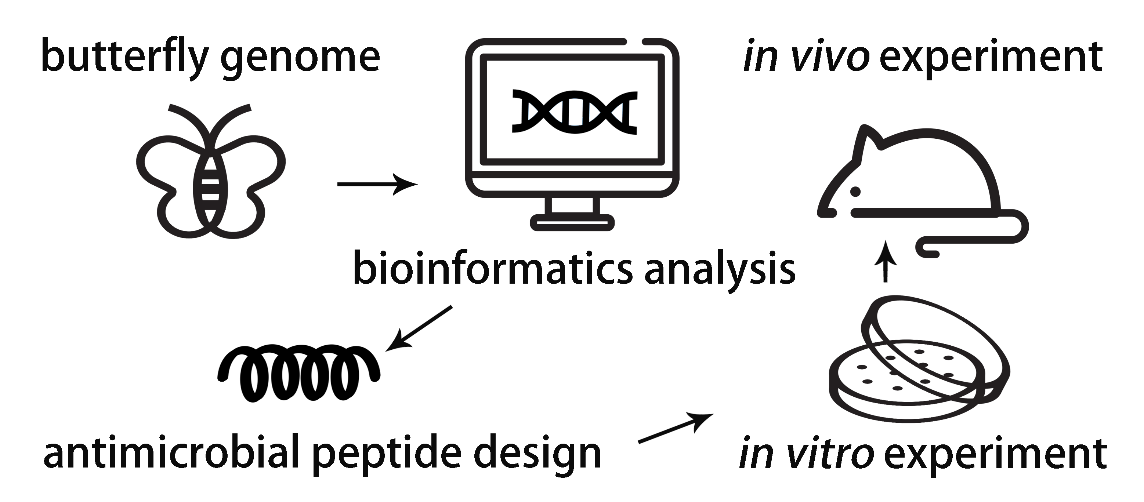
**

**Fig. S1 Schematic illustration of the experimental design.**


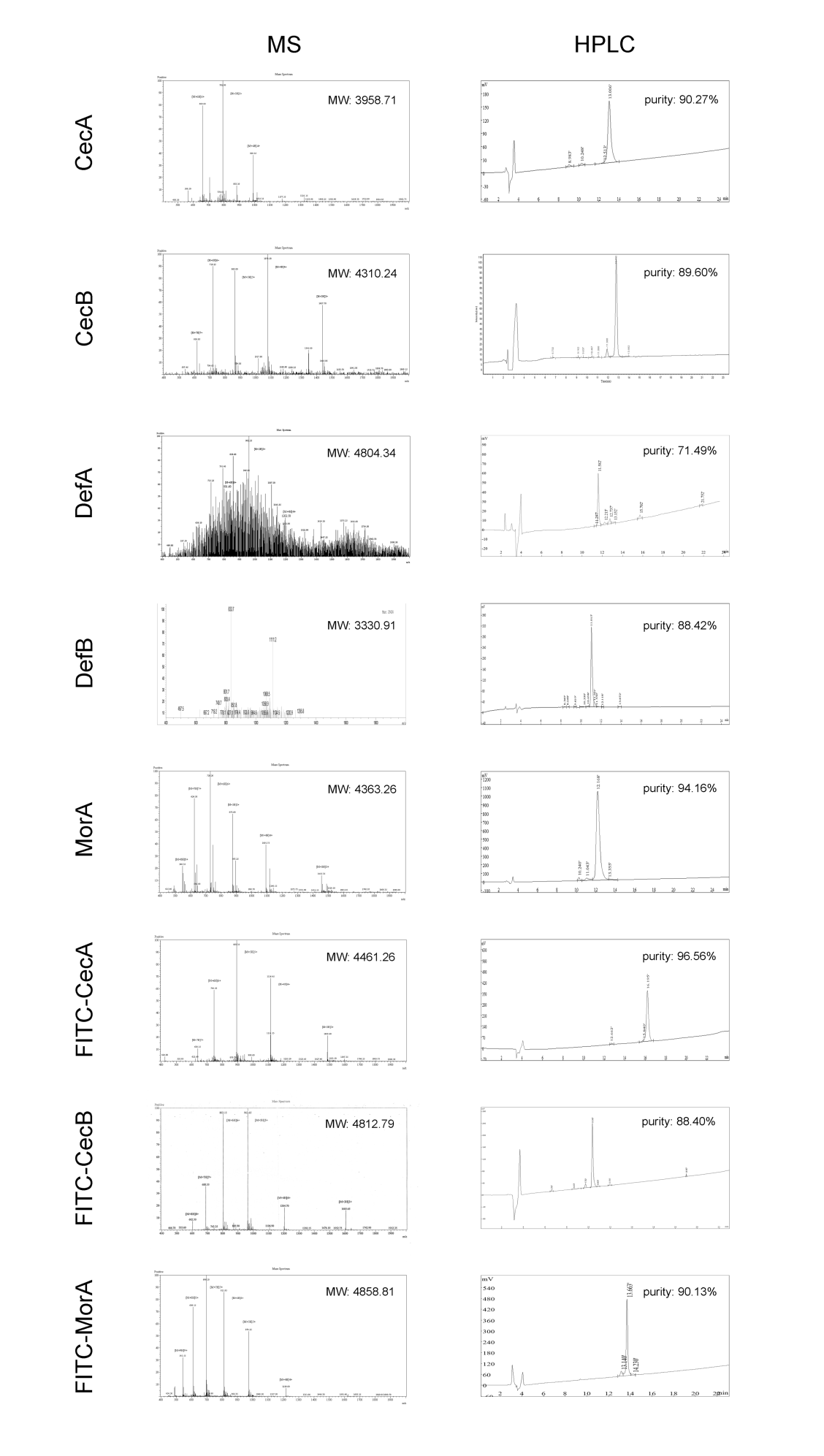


**Fig. S2 HPLCprofiles and spectrometric data of the synthetic AMPs.**


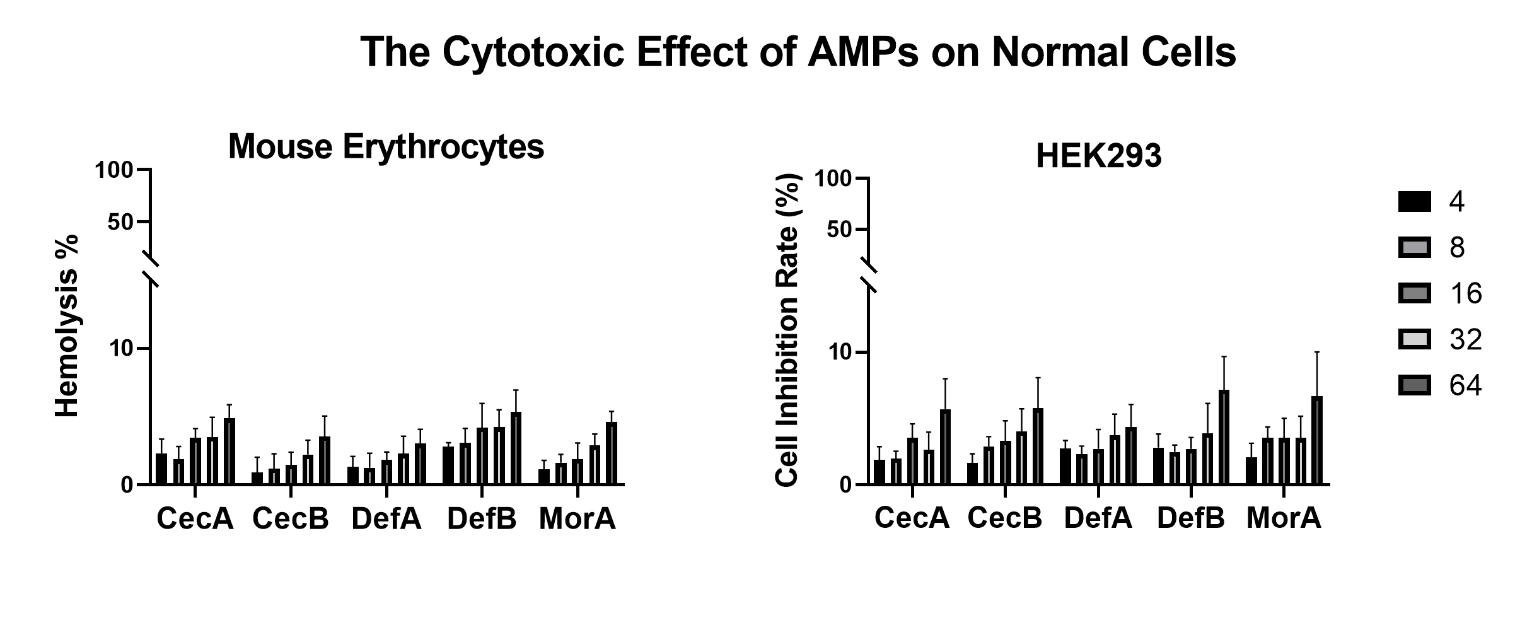
**Figure S3. Evaluation of hemolytic and cytotoxic activities of synthetic AMPs.** The hemolysis effects (left) and cytotoxicityon normal cells (right) of the synthetic peptides at different concentration were tested on mice erythrocytes and HEK293 cells, respectively. PBS and Triton-X100 were used as the negative and positive control. No apparent hemolysis or cytotoxicity was detected among groups.


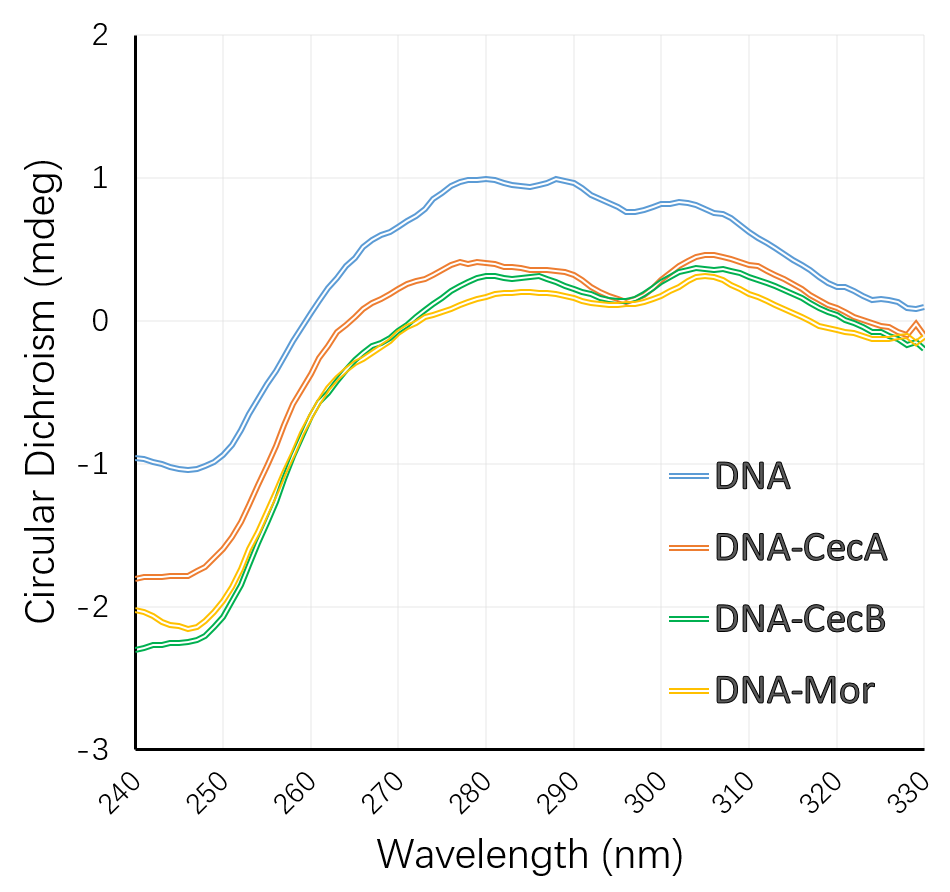


**Fig. S4 CD spectrum of the designed AMPs interacted with fungal genomic DNA.** The top curve corresponded to free DNA, while the lower curves were that mixed with respective AMPs. The decreased intensities of genomic DNA molar ellipticity exhibited occurrence conformational alteration of DNA that induced by AMPs, which suggested a DNA-binding mechanism of the designed sequences.

**
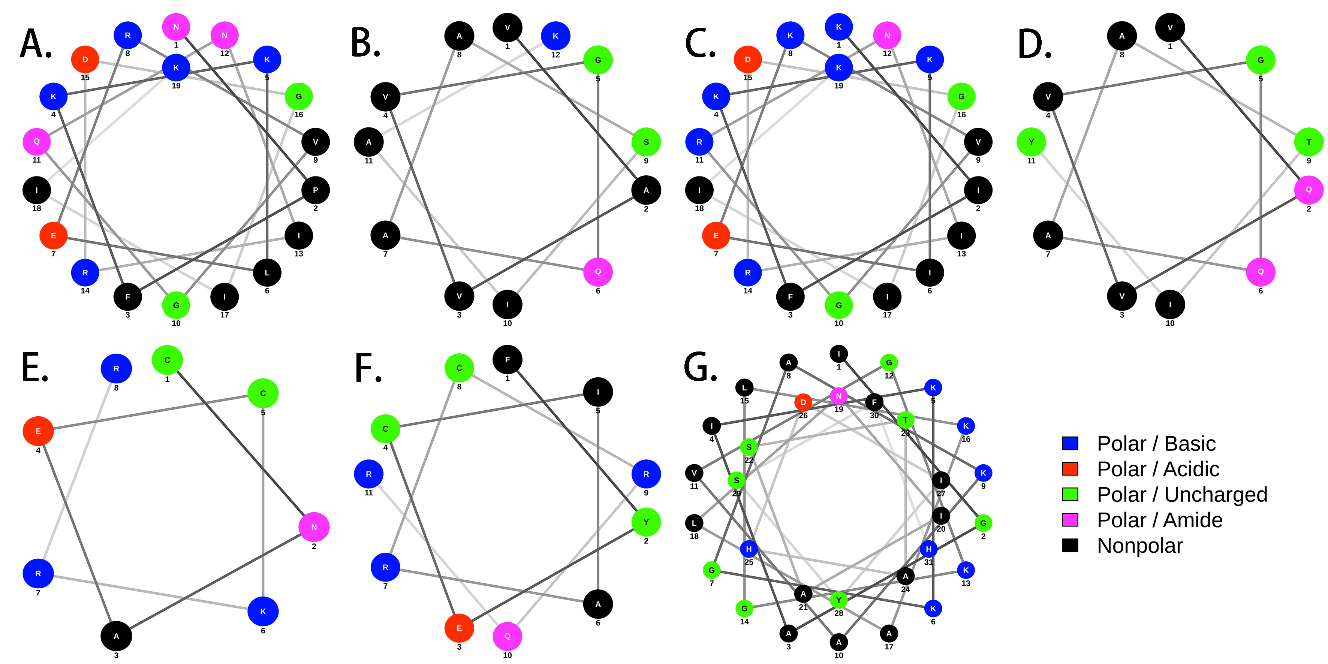
**

**Fig. S5 Helical wheel projection analysis of the designed peptide.** (**A**) N-terminal amphiphilic helix of cecropin A; (**B**) C-terminal hydrophobic helix of cecropin A; (**C**) N-terminal amphiphilic helix of cecropin B; (**D**) C-terminal hydrophobic helix of cecropin B; (**E**) helical segment of defensin A; (**F**) helical segment of defensin B; (**G**) helical segment of moricin A.

**Tab. S1 Papilionoidea species andtheir high-throughput sequencing data used in this study**

| **Family** | **Taxa** | **Taxon ID** | **Accession No.** |
| --- | --- | --- | --- |
| *Hesperiidae* | *Lerema accius* | 691651 | GCA_001278395.1 |
| *Lycaenidae* | *Calycopis cecrops* | 691633 | GCA_001625245.1 |
| *Nymphalidae* | *Bicyclus anynana* | 110368 | GCA_900239965.1 |
| *Nymphalidae* | *Danaus plexippus* | 13037 | GCA_000235995.2 |
| *Nymphalidae* | *Heliconius cydno* | 33424 | GCA_900068235.1 |
| *Nymphalidae* | *Heliconius elevatus* | 33444 | GCA_900068365.1 |
| *Nymphalidae* | *Heliconius ethilla* | 33418 | GCA_001485985.1 |
| *Nymphalidae* | *Heliconius hecale* | 33425 | GCA_001486065.1 |
| *Nymphalidae* | *Heliconius hecuba* | 101931 | GCA_900068455.1 |
| *Nymphalidae* | *Heliconius heurippa* | 33423 | GCA_900067975.1 |
| *Nymphalidae* | *Heliconius hierax* | 171913 | GCA_900068475.1 |
| *Nymphalidae* | *Heliconius ismenius* | 33427 | GCA_001485965.1 |
| *Nymphalidae* | *Heliconius melpomene* | 34740 | GCA_000313835.2 |
| *Nymphalidae* | *Heliconius numata* | 33419 | GCA_900068715.1 |
| *Nymphalidae* | *Heliconius pachinus* | 33428 | GCA_900068735.1 |
| *Nymphalidae* | *Heliconius pardalinus* | 33441 | GCA_001486225.1 |
| *Nymphalidae* | *Heliconius timareta* | 101932 | GCA_900068805.1 |
| *Nymphalidae* | *Heliconius wallacei* | 33429 | GCA_900068815.1 |
| *Nymphalidae* | *Heliconius xanthocles* | 33440 | GCA_900068825.1 |
| *Nymphalidae* | *Laparus doris* | 33455 | GCA_900068335.1 |
| *Nymphalidae* | *Melitaea cinxia* | 113334 | GCA_000716385.1 |
| *Nymphalidae* | *Neruda aoede* | 33457 | GCA_900068225.1 |
| *Nymphalidae* | *Vanessa tameamea* | 334116 | GCA_002938995.1 |
| *Papilionidae* | *Atrophaneura mencius* | 199695 | GSE142679 |
| *Papilionidae* | *Papilio clytia* | 110796 | GSE142679 |
| *Papilionidae* | *Papilio glaucus* | 45779 | GCA_000931545.1 |
| *Papilionidae* | *Papilio machaon* | 76193 | GCF_001298355.1 |
| *Papilionidae* | *Papilio memnon* | 76198 | GCA_003118335.1 |
| *Papilionidae* | *Papilio polytes* | 76194 | GCF_000836215.1 |
| *Papilionidae* | *Papilio xuthus* | 66420 | GCF_000836235.1 |
| *Pieridae* | *Leptidea sinapis* | 189913 | GCA_900199415.1 |
| *Pieridae* | *Phoebis sennae* | 40077 | GCA_001586405.1 |
| *Pieridae* | *Pieris rapae* | 64459 | GCF_001856805.1 |
| *Riodinidae* | *Calephelis nemesis* | 2010996 | GCA_002245505.1 |
| *Riodinidae* | *Calephelis virginiensis* | 2010997 | GCA_002245475.1 |

**Tab. S2 The AMP sequences for phylogenetic analysis in this study**

|  |  | **Source species** | **AMP name** | **Mature peptide sequence** |
| --- | --- | --- | --- | --- |
| ***Cecropins*** | | | | |
|  | 1 | *Lerema accius* | Cecropin 1 | RWNPFKKLERVGQNIRDGIIKAGPAVAVVGQAASIYKGK |
|  | 2 | *Lerema accius* | Cecropin 2 | RWNPFKKLEKVGQNIRDGIIKAGPAVAVVGEAANIYKGK |
|  | 3 | *Neruda aoede* | Cecropin 3 | WNPFKELEKAGQRVRDAIISAGPAVQVVGQATSIIKGGN |
|  | 4 | *Vanessa tameamea* | Cecropin 4 | KWKLFKRIEKIGRNVRNGLIKAGPAIQVVGQA |
|  | 5 | *Pieris rapae* | Cecropin 5 | KWKIFKKIEHFGQNIRDGLIKAGPALQVVGEAATIYKGK |
|  | 6 | *Calycopis cecrops* | Cecropin 6 | WNPLKELERAGQRVRDAIISAGPAVDVVEKTAAIIKGGQQ |
|  | 7 | *Papilio xuthus* | Cecropin 7 | WNPFKELERAGQNIRDAIISAGPAVDVVARAQKIARGEDVDEDD |
|  | 8 | *Lerema accius* | Cecropin 8 | RWNPFKKLERVGQNIRDGIIKAGPASRCGGGPSREHIQGKMNLSV |
|  | 9 | *Papilio machaon* | Cecropin 9 | RWNPFKKLEKVGQNIRDGIIKAGPAVEVIGQAASIVKPNQGK |
|  | 10 | *Papilio machaon* | Cecropin 10 | KWKIFKKIEKVGRNVRDGIIKAGPAVAVVGQAATVAKG |
|  | 11 | *Papilio machaon* | Cecropin 11 | KWKLFKKIEKVGRNIRNGIIKAGPAVQVVGQASQIYKQG |
|  | 12 | *Papilio machaon* | Cecropin 12 | WNPFKELERAGQNIRDAIISAGPAVDVVARAQKIARGEDVDEEE |
|  | 13 | *Papilio xuthus* | Cecropin 13 | RWKIFKKIEKVGRNVRDGIIKAGPAVAVVGQAATVVKG |
|  | 14 | *Papilio polytes* | Cecropin 14 | KWKIFKKIERVGQNIRDGIIKAGPAVAVVGQAASIIKPGK |
|  | 15 | *Danaus plexippus* | Cecropin 15 | KWKPFKKLEKIGQRVRDGIIKAGPAVQVVGEAAAILKPAQG |
|  | 16 | *Danaus plexippus* | Cecropin 16 | RWKFLKKIEKVGRKVRDGVIKAGPAVGVVGQATSIYKGK |
|  | 17 | *Danaus plexippus* | Cecropin 17 | KWKIFKKIEKVGRNVRDGIIKAGPAVQVVGQATSIAKG |
|  | 18 | *Danaus plexippus* | Cecropin 18 | KWKFFKKIEKVGRNIRDGIIKAGPAVQVLGEAKAIGK |
|  | 19 | *Pieris rapae* | Cecropin 19 | KWKIFKKIEHMGQNIRDGLIKAGPAVQVVGQAATIYKG |
|  | 20 | *Vanessa tameamea* | Cecropin 20 | KWKLFKRIEKLGQRVRDGIIKAGPAVGVIGQASTIIKG |
|  | 21 | *Pieris rapae* | Cecropin 21 | KWKIFKKIEHMGQNIRDGLIKAGPAVQVVGQAATIYKGK |
|  | 22 | *Pieris rapae* | Cecropin 22 | KWKIFKKIEHMGQNIRDGLIKAGPAVQVVGEAATIYKGK |
|  | 23 | *Danaus plexippus* | Cecropin 23 | WNPFKELEKAGQRVRDAIISAAPAVEVVGQASSILKGKN |
|  | 24 | *Papilio xuthus* | Cecropin 24 | WNPFKELERAGQNIRDAIISAGPAVDVVARAQKIARGEDVDEDE |
|  | 25 | *Papilio xuthus* | Cecropin 25 | KWKLFKKIEKVGRNIRNGIIKAGPAVQVVGQASQIYKLG |
|  | 26 | *Papilio xuthus* | Cecropin 26 | RWNPFKKLERVGQNIRDGIIKAGPAVAVVGQAASIIKPGK |
|  | 27 | *Papilio xuthus* | Cecropin 27 | KWNPFKKLEKVGQNIRDGIIKAGPAVQVIGQAASIVKPNQGK |
|  | 28 | *Papilio xuthus* | Cecropin 28 | RWKIFKKIEKVGRNVRDGIIKAGPAVAVVEQAATVVKG |
|  | 29 | *Papilio clytia* | Cecropin 29 | RWKLFKKIEKVGRNIRDGIIKAGPAVQVVGQASQIYKLG |
|  | 30 | *Atrophaneura mencius* | Cecropin 30 | RWNPFKKLEKVGQNIRDGIVKAGPAVGVIGQAASIVKPGK |
|  | 31 | *Hyalophora cecropia* | Cecropin B | KWKVFKKIEKMGRNIRNGIVKAGPAIAVLGEAKAILS |
|  | 32 | *Heliothis virescens* | Cecropin A | RWKVFKKIEKVGRNIRDGVIKAAPAIEVLGQAKAL |
|  | 33 | *Antheraea pernyi* | Cecropin B | KWKIFKKIEKVGRNIRNGIIKAGPAVAVLGEAKAL |
|  | 34 | *Antheraea pernyi* | Cecropin D | WNPFKELERAGQRVRDAIISAGPAVATVAQATALAK |
|  | 35 | *Hyalophora cecropia* | Cecropin A | KWKLFKKIEKVGQNIRDGIIKAGPAVAVVGQATQIAK |
|  | 36 | *Bombyx mori* | Cecropin B | RWKIFKKIEKMGRNIRDGIVKAGPAIEVLGSAKAI |
|  | 37 | *Plutella xylostella* | Cecropin A1 | RWKPFKKELKVGRNIRDGIIKAGPAVAVIGQATSIARPTGK |
|  | 38 | *Bombyx mori* | Cecropin XJ | RWKIFKKIEKMGRNIRDGIVKAGPAIEVLGSAKAIGK |
|  | 39 | *Galleria mellonella* | Cecropin A | KWKIFKKIEKAGRNIRDGIIKAGPAVSVVGEAATIYKTG |
|  | 40 | *Galleria mellonella* | Cecropin B | KWKFFKKIERVGQNIRDGIIKAGPAVQVVGQAATIYKGK |
|  | 41 | *Galleria mellonella* | Cecropin C | RWKVFKKIERMGQHIRDGIIKAGPAVAVVGQASTIISG |
|  | 42 | *Heliothis virescens* | Cecropin B | KWKVFKKIEKVGRNIRDGIVKAGPAIAVLGQAN |
|  | 43 | *Heliothis virescens* | Cecropin C | RWKVFKKIEKMGRNIRDGVIKAAPAIEVLGQAK |
|  | 44 | *Oiketicus kirbyi* | Cecropin | WKPFKKIEKAVRRVRDGVAKAGPAVAVVGQAT |
|  | 45 | *Bombyx mori* | Cecropin | RWKIFKKIEKVGQNIRDGIVKAGPAVAVVGQAATI |
|  | 46 | *Aedes albopictus* | Cecropin A1 | GGLKKLGKKLEGVGKRVFKASEKALPVAVGIKALGK |
|  | 47 | *Glossina morsitans* | Cecropin | GWLKKIGKKIERVGQNTRDATVKGLEVAQQAANVAATVR |
|  | 48 | *Glossina morsitans* | Cecropin C | GWLKKLGKRIERIGQHTRDATIQGLGIAQQAANVAATAR |
|  | 49 | *Ceratitis capitata* | Cecropin 1 | GWLKKIGKKIERVGQHTRDATIQTIAVAQQAANVAATAR |
|  | 50 | *Aedes albopictus* | Cecropin B | GGLKKLGKKLEGVGKRVFKASEKALPVLTGYKAIG |
|  | 51 | *Musca domestica* | Cecropin 2 | GWLKKIGKKIERVGQHTRDATIQTIGVAQQAANVAATLK |
|  | 52 | *Drosophila melanogaster* | Cecropin A2 | GWLKKIGKKIERVGQHTRDATIQGLGIAQQAANVAATAR |
|  | 53 | *Aedes albopictus* | Cecropin C | GGLKKLGKKLEGAGKRVFNAAEKALPVVAGAKALG |
|  | 54 | *Drosophila melanogaster* | Cecropin A2 | GWLRKLGKKIERIGQHTRDASIQVLGIAQQAANVAATAR |
|  | 55 | *Lucilia sericata* | Cecropin 1 | GWLKKIGKKIERVGQHTRDATIQTIGVAQQAANVAATLKG |
|  | 56 | *Lucilia sericata* | Cecropin 2 | GWLRDFGKRIERVGQHTRDATIQAIGVAQQAANVAATVRG |
|  | 57 | *Lucilia sericata* | Cecropin 3 | GWLKKIGKKIERVGQHTRDATIQVLGVAQQAANVAATARG |
|  | 58 | *Lucilia sericata* | Cecropin 4 | GWLKKIGKKIERVGQHTRDASIQAIGIAQQAANVAATARG |
|  | 59 | *Lucilia sericata* | Cecropin 5 | GLVKKIGKKIERVGQHTRDASIQAIGIAQQAANVAATARG |
|  | 60 | *Lucilia sericata* | Cecropin 6 | GWLKKFGKKIERVGQHTRDATIQAIGVAQQAANVAATLKG |
|  | 61 | *Calliphora vicina* | Cecropin | GWLKKIGKKIGRVGQHTRDATIQGLAVAQQAANVAATAR |
| ***Defensins*** | | | | |
|  | 1 | *Calycopis cecrops* | Defensin 1 | DKLIGSCVWGAVNYTSNCNAECKRRGYKGGHCGSFANVNCWCET |
|  | 2 | *Lerema accius* | Defensin 2 | DKLIGSCVWGAVNYTSDCNAECKRRGYRGGHCGSFANVNCWCET |
|  | 3 | *Phoebis sennae* | Defensin 3 | DKLIGSCVWGAVNYTSDCNKECKRRGYKGGHCGSFANVNCWCET |
|  | 4 | *Papilio polytes* | Defensin 4 | GCVFYECIARCRQRGYLSGGYCTINGCQCLG |
|  | 5 | *Papilio memnon* | Defensin 5 | GCVFYECIARCRQRGYLSGGYCTINGCQCLG |
|  | 6 | *Papilio clytia* | Defensin 6 | GCIFYECIARCRQRGHLSGGYCTINGCQCLG |
|  | 7 | *Galleria mellonella* | Defensin | DKLIGSCVWGATNYTSDCNAECKRRGYKGGHCGSFWNVNCWCEE |
|  | 8 | *Galleria mellonella* | Defensin | DTLIGSCVWGATNYTSDCNAECKRRGYKGGHCGSFLNVNCWCE |
|  | 9 | *Heliothis virescens* | heliomicin | DKLIGSCVWGAVNYTSDCNGECKRRGYKGGHCGSFANVNCWCET |
|  | 10 | *Helicoverpa zea* | Defensin | DKLIGSCVWGAVNYTSDCNGECKRRGYKGGHCGSFANVNCWGET |
|  | 11 | *Thitarodes jiachaensis* | Defensin | APCDLFSFLGVHGFECAVHCLSMFKGFTGGYCADGVCICRK |
|  | 12 | *Hepialus xiaojinensis* | Defensin | ATCDLFSGLGVEDSLCAAHCLAMFKGFRGGHCVDGVCICRK |
|  | 13 | *Drosophila melanogaster* | Defensin | ATCDLLSKWNWNHTACAGHCIAKGFKGGYCNDKAVCVCRN |
|  | 14 | *Bombus pascuorum* | Defensin | VTCDLLSIKGVAEHSACAANCLSMGKAGGRCENGICLCRKTTFKELWDKRF |
|  | 15 | *Cimex lectularius* | Defensin | ATCDLFSFQSKWVTPNHAACAAHCTARGNRGGRCKKAVCHCRK |
|  | 16 | *Aeshna cyanea* | Defensin | GFGCPLDQMQCHRHCQTITGRSGGYCSGPLKLTCTCYR |
|  | 17 | *Tenebrio molitor* | Tenecin 1 | VTCDILSVEAKGVKLNDAACAAHCLFRGRSGGYCNGKRVCVCR |
|  | 18 | *Aedes aegypti* | Defensin A | ATCDLLSGFGVGDSACAAHCIARGNRGGYCNSKKVCVCRN |
|  | 19 | *Leiurus quinquestriatus* | defensin | GFGCPLNQGACHRHCRSIRRRGGYCAGFFKQTCCYRN |
|  | 20 | *Androctonus australis* | defensin | GFGCPFNQGACHRHCRSIRRRGGYCAGLFKQTCTCYR |
|  | 21 | *Ornithodoros moubata* | Defensin-A | GYGCPFNQYQCHSHCSGIRGYKGGYCKGTFKQTCKCY |
|  | 22 | *Ixodes scapularis* | Scapularisin-6 | GFGCPFDQGACHRHCQSIGRRGGYCAGFIKQTCTCYHN |
|  | 23 | *Pseudoplectania nigrella* | Plectasin | GFGCNGPWDEDDMQCHNHCKSIKGYKGGYCAKGGFVCKCY |
|  | 24 | *Eurotium amstelodami* | Eurocin | GFGCPGDAYQCSEHCRALGGGRTGGYCAGPWYLGHPTCTCSF |
|  | 25 | *Microsporum canis* | Micasin-1 | GFGCPFNENECHAHCLSIGRKFGFCAGPLRATCTCGKQ |
|  | 26 | *Trichophyton interdigitale* | Triintsin | GFGCPLNERECHSHCQSIGRKFGYCGGTLRLTCICGKE |
|  | 27 | *Mytilus edulis* | Mytilin | GFGCPNDYPCHRHCKSIPGRAGGYCGGAHRLRCTCYR |
|  | 28 | *Crassostrea gigas* | Cg-Def | GFGCPGNQLKCNNHCKSISCRAGYCDAATLWLRCTCTDCNGKK |
|  | 29 | *Crassostrea virginica* | AOD | GFGCPWNRYQCHSHCRSIGRLGGYCAGSLRLTCTCYRS |
|  | 30 | *Mytilus galloprovincialis* | MGD-1 | GFGCPNNYQCHRHCKSIPGRCGGYCGGWHRLPCTCYRCG |
|  | 31 | *Sparus aurata* | saBD | ASFPWSCPSLSGVCRKVCLPTELFFGPLGCGKGFLCGVSHFL |
|  | 32 | *Gadus morhua* | Defensin | WSCPTLSGVCRKVCLPTEMFFGPLGCGKEFQCCVSHFF |
|  | 33 | *Oreochromis niloticus* | Defensin | FPWSCLSLSGVCRKVCLPTELFFGPLGCGKGSLCCVSHFL |
|  | 34 | *Ictalurus punctatus* | ccBD | VSFPWSCAALSGVCRQGACLPSELYFGPLGCGKGSLCCVSYFL |
|  | 35 | *Polypedates puerensis* | PopuDef | GASPALWGCDSFLGYCRIACFAHEASVGQKDCAEGMICCLPNVF |
|  | 36 | *Theloderma kwangsiensis* | Defensin-TK | SPAIWGCDSFLGYCRLACFAHEASVGQKECAEGMLCCIPNV |
|  | 37 | *Cynops fudingensis* | CFBD-1 | FAVWGCADYRGYCRAACFAFEYSLGPKGCTEGYVCCVPNTF |
|  | 38 | *Rhacophorus puerensis* | Defensin | SPALWGCDSFLGYCRIACFAHEASVGQKDCAEGMICCLPNVF |
|  | 39 | *Aptenodytes patagonicus* | Spheniscin-2 | SFGLCRLRRGFCARGRCRFPSIPIGRCSRFVQCCRRVW |
|  | 40 | *Gallus gallus* | Gal-1a | GRKSDCFRKNGFCAFLKCPYLTLISGKCSRFHLCCKRIW |
|  | 41 | *Struthio camelus* | Ostricacin-2 | APGNKAECEREKGYCGFLKCSFPFVVSGKCSRFFFCCKNIW |
|  | 42 | *Anas platyrhynchos* | AvBD-9 | ADTLACRQSHQSCSFVACRAPSVDIGTCRGGKLKCCKWAPSS |
|  | 43 | *Mus musculus* | Cryptdin-1 | LRDLVCYCRTRGCKRRERMNGTCRKGHLMYTLCCR |
|  | 44 | *Homo sapien* | HD-5 | ATCYCRTGRCATRESLSGVCEISGRLYRLCCR |
|  | 45 | *Mesocricetus auratus* | HANP-1 | VTCFCRRRGCASRERHIGYCRFGNTIYRLCCRR |
| ***Moricins*** | | | | |
|  | 1 | *Danaus plexippus* | Moricin 1 | ARIPIGAIRKGAKAVGKGLRAINIAGTVHDIVEVFKPRKRKH |
|  | 2 | *Calycopis cecrops* | Moricin 2 | GKIPIGAIKKGAKLVGKGLKALNIASTANDVYHFFHHKRKH |
|  | 3 | *Calycopis cecrops* | Moricin 3 | GKIPIGAIKKGAEVVGKGLKALNIASTANDVYKFFHHKKKH |
|  | 4 | *Danaus plexippus* | Moricin 4 | GKIPINAIRKGAKAVGHGLRALNIASTAHDIVSAFKHKKRKH |
|  | 5 | *Papilio polytes* | Moricin 5 | GIPIGAIKKGGQWIRKGFGVLSAAGTAHEVYSHVKNRRN |
|  | 6 | *Pieris rapae* | Moricin 6 | GKIPKAVIKKGAKLVGNGLKALNVASTVHDIYSALHHKKKKH |
|  | 7 | *Bicyclus anynana* | Moricin 7 | KIPINAIRKGARAVGKGLRMINYASTAHDIASMFHKKKRKH |
|  | 8 | *Bombyx mori* | Moricin | AKIPIKAIKTVGKAVGKGLRAINIASTANDVFNFLKPKKRKA |
|  | 9 | *Bombyx mori* | Moricin 2 | AKIPIKAIKTVGKAVGKGLRAINIASTANDVFNFLKPKKRKH |
|  | 10 | *Spodoptera litura* | Moricin | GKIPVKAIKKAGAAIGKGLRAINIASTAHDVYSFFKPKHKKK |
|  | 11 | *Galleria mellonella* | Gm-mlpA | KVNANAIKKGGKAIGKGFKVISAASTAHDVYEHIKNRRH |
|  | 12 | *Galleria mellonella* | Gm-mlpB | GKIPVKAIKKGGQIIGKALRGINIASTAHDIISQFKPKKKKNH |
|  | 13 | *Galleria mellonella* | Gm-mlpC1 | KVPIGAIKKGGKIIKKGLGVIGAAGTAHEVYSHVKNRH |
|  | 14 | *Galleria mellonella* | Gm-mlpC2 | KVPIGAIKKGGKIIKKGLGVLGAAGTAHEVYNHVRNRQ |
|  | 15 | *Galleria mellonella* | Gm-mlpC3 | KVPIGAIKKGGKIIKKGLGVIGAAGTAHEVYSHVKNRQ |
|  | 16 | *Galleria mellonella* | Gm-mlpC4/C5 | KVPVGAIKKGGKAIKTGLGVVGAAGTAHEVYSHIRNRH |
|  | 17 | *Galleria mellonella* | Gm-mlpD | KGIGSALKKGGKIIKGGLGALGAIGTGQQVYEHVQNRQ |
|  | 18 | *Manduca sexta* | Moricin | GKIPVKAIKQAGKVIGKGLRAINIAGTTHDVVSFFRPKKKKH |
|  | 19 | *Plutella xylostella* | Px-Mor | APKVNVNALKKGGRVIKKGLGVIGAAGTAHEVYNHVRNRNQG |
|  | 20 | *Heliothis virescens* | Virescein | GKIPIGAIKKAGKAIGKGLRAVNIASTAHDVYTFFKPKKRH |
|  | 21 | *Helicoverpa armigera* | Moricin | GKIPVGAIKKAGRAIGKGLRAINIASTAHDVYTFFKPKKRH |

**Tab. S3 Sequence alignment of the synthetic and naturally occurring AMPs**

| **AMPs** | **Sequences** |
| --- | --- |
|  |  |
| **CecA** | RWNPFKKLERVGQNIRDGIIKAGPAVAVVGQAASIAK-------- |
| Cec01 | RWNPFKKLERVGQNIRDGIIKAGPAVAVVGQAASIYKGK------ |
| Cec02 | RWNPFKKLEKVGQNIRDGIIKAGPAVAVVGEAANIYKGK------ |
| Cec03 | -WNPFKELEKAGQRVRDAIISAGPAVQVVGQATSIIKGGN----- |
| Cec06 | -WNPLKELERAGQRVRDAIISAGPAVDVVEKTAAIIKGGQQ---- |
| Cec07 | -WNPFKELERAGQNIRDAIISAGPAVDVVARAQKIARGEDVDEDD |
| Cec08 | RWNPFKKLERVGQNIRDGIIKAGPASRCGGGPSREHIQGKMNLSV |
| Cec09 | RWNPFKKLEKVGQNIRDGIIKAGPAVEVIGQAASIVKPNQGK--- |
| Cec12 | -WNPFKELERAGQNIRDAIISAGPAVDVVARAQKIARGEDVDEEE |
| Cec23 | -WNPFKELEKAGQRVRDAIISAAPAVEVVGQASSILKGKN----- |
| Cec24 | -WNPFKELERAGQNIRDAIISAGPAVDVVARAQKIARGEDVDEDE |
| Cec26 | RWNPFKKLERVGQNIRDGIIKAGPAVAVVGQAASIIKPGK----- |
| Cec27 | KWNPFKKLEKVGQNIRDGIIKAGPAVQVIGQAASIVKPNQGK--- |
| Cec30 | RWNPFKKLEKVGQNIRDGIVKAGPAVGVIGQAASIVKPGK----- |
|  |  |
| **CecB** | KWKIFKKIEKVGRNIRDGIIKAGPAVQVVGQAATIYKGK-- |
| Cec04 | KWKLFKRIEKIGRNVRNGLIKAGPAIQVVGQA--------- |
| Cec05 | KWKIFKKIEHFGQNIRDGLIKAGPALQVVGEAATIYKGK-- |
| Cec10 | KWKIFKKIEKVGRNVRDGIIKAGPAVAVVGQAATVAKG--- |
| Cec11 | KWKLFKKIEKVGRNIRNGIIKAGPAVQVVGQASQIYKQG-- |
| Cec13 | RWKIFKKIEKVGRNVRDGIIKAGPAVAVVGQAATVVKG--- |
| Cec14 | KWKIFKKIERVGQNIRDGIIKAGPAVAVVGQAASIIKPGK- |
| Cec15 | KWKPFKKLEKIGQRVRDGIIKAGPAVQVVGEAAAILKPAQG |
| Cec16 | RWKFLKKIEKVGRKVRDGVIKAGPAVGVVGQATSIYKGK-- |
| Cec17 | KWKIFKKIEKVGRNVRDGIIKAGPAVQVVGQATSIAKG--- |
| Cec18 | KWKFFKKIEKVGRNIRDGIIKAGPAVQVLGEAKAIGK---- |
| Cec19 | KWKIFKKIEHMGQNIRDGLIKAGPAVQVVGQAATIYKG--- |
| Cec20 | KWKLFKRIEKLGQRVRDGIIKAGPAVGVIGQASTIIKG--- |
| Cec21 | KWKIFKKIEHMGQNIRDGLIKAGPAVQVVGQAATIYKGK-- |
| Cec22 | KWKIFKKIEHMGQNIRDGLIKAGPAVQVVGEAATIYKGK-- |
| Cec25 | KWKLFKKIEKVGRNIRNGIIKAGPAVQVVGQASQIYKLG-- |
| Cec28 | RWKIFKKIEKVGRNVRDGIIKAGPAVAVVEQAATVVKG--- |
| Cec29 | RWKLFKKIEKVGRNIRDGIIKAGPAVQVVGQASQIYKLG-- |
|  |  |
| **DefA** | DKLIGSCVWGAVNYTSDCNAECKRRGYKGGHCGSFANVNCWCET |
| Def01 | DKLIGSCVWGAVNYTSNCNAECKRRGYKGGHCGSFANVNCWCET |
| Def02 | DKLIGSCVWGAVNYTSDCNAECKRRGYRGGHCGSFANVNCWCET |
| Def03 | DKLIGSCVWGAVNYTSDCNKECKRRGYKGGHCGSFANVNCWCET |
|  |  |
| **DefB** | GCVFYECIARCRQRGYLSGGYCTINGCQCL- |
| Def04 | GCVFYECIARCRQRGYLSGGYCTINGCQCLG |
| Def05 | GCVFYECIARCRQRGYLSGGYCTINGCQCLG |
| Def06 | GCIFYECIARCRQRGHLSGGYCTINGCQCLG |
|  |  |
| **MorA** | GKIPIGAIKKGAKAVGKGLKALNIASTAHDIYSFHHKKKKH- |
| Mor01 | ARIPIGAIRKGAKAVGKGLRAINIAGTVHDIVEVFKPRKRKH |
| Mor02 | GKIPIGAIKKGAKLVGKGLKALNIASTANDVYHFFHHKRKH- |
| Mor03 | GKIPIGAIKKGAEVVGKGLKALNIASTANDVYKFFHHKKKH- |
| Mor04 | GKIPINAIRKGAKAVGHGLRALNIASTAHDIVSAFKHKKRKH |
| Mor05 | -GIPIGAIKKGGQWIRKGFGVLSAAGTAHEVYSHVKNRRN-- |
| Mor06 | GKIPKAVIKKGAKLVGNGLKALNVASTVHDIYSALHHKKKKH |
| Mor07 | -KIPINAIRKGARAVGKGLRMINYASTAHDIASMFHKKKRKH |
